# Optogenetic Stimulation of mPFC Alleviates White Matter Injury‐Related Cognitive Decline after Chronic Ischemia through Adaptive Myelination

**DOI:** 10.1002/advs.202202976

**Published:** 2022-12-18

**Authors:** Shiji Deng, Shu Shu, Lili Zhai, Shengnan Xia, Xiang Cao, Huiya Li, Xinyu Bao, Pinyi Liu, Yun Xu

**Affiliations:** ^1^ Department of Neurology Drum Tower Hospital Medical School and The State Key Laboratory of Pharmaceutical Biotechnology Institute of Translational Medicine for Brain Critical Diseases Nanjing University Nanjing 210008 China; ^2^ Jiangsu Key Laboratory for Molecular Medicine Medical School of Nanjing University Nanjing 210008 China; ^3^ Jiangsu Provincial Key Discipline of Neurology Nanjing 210008 China; ^4^ Nanjing Neurology Medical Center Nanjing 210008 China

**Keywords:** adaptive myelination, chemogenetics, chronic ischemia, optogenetics, Wnt2

## Abstract

White matter injury (WMI), which reflects myelin loss, contributes to cognitive decline or dementia caused by cerebral vascular diseases. However, because pharmacological agents specifically for WMI are lacking, novel therapeutic strategies need to be explored. It is recently found that adaptive myelination is required for homeostatic control of brain functions. In this study, adaptive myelination‐related strategies are applied to explore the treatment for ischemic WMI‐related cognitive dysfunction. Here, bilateral carotid artery stenosis (BCAS) is used to model ischemic WMI‐related cognitive impairment and uncover that optogenetic and chemogenetic activation of glutamatergic neurons in the medial prefrontal cortex (mPFC) promote the differentiation of oligodendrocyte precursor cells (OPCs) in the corpus callosum, leading to improvements in myelin repair and working memory. Mechanistically, these neuromodulatory techniques exert a therapeutic effect by inducing the secretion of Wnt2 from activated neuronal axons, which acts on oligodendrocyte precursor cells and drives oligodendrogenesis and myelination. Thus, this study suggests that neuromodulation is a promising strategy for directing myelin repair and cognitive recovery through adaptive myelination in the context of ischemic WMI.

## Graphical abstract

Optogenetic and chemogenetic activation of mPFC glutamatergic neurons after chronic ischemia upregulate the expression of neuron‐derived Wnt2, which acts on OPCs and promotes their differentiation toward oligodendrocytes, leading to myelin repair and improved cognitive function. The current finding highlights that neuromodulation is a promising strategy for directing adaptive myelination in the context of chronic ischemia.

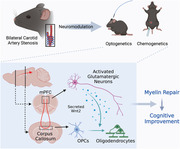

## 1 Introduction

White matter injury (WMI), which can be visualized as hyperintensity on T2‐weighted imaging or FLAIR on magnetic resonance imaging (MRI), is a common pathological feature of cerebral vascular disease.^[^ ^1^ ^]^ White matter hyperintensity (WMH) is observed in ≈50% of middle‐aged individuals and 90–100% of elderly individuals.^[^ ^2^ ^]^ It has been reported that WMH is the most common lesion type in cerebral small vessel disease (CSVD) and is associated with poor prognosis of ischemic stroke in patients with carotid artery stenosis, indicating its contributions to both small and large vessel diseases.^[^ ^3^ ^]^ In addition, the WMH burden may cause cognitive decline, especially impairment of executive functions.^[^ ^4^ ^]^ Specifically, WMH is associated with a 73% increased risk of vascular dementia (VaD) and a 25% increased risk of Alzheimer's disease (AD).^[^ ^5^ ^]^ Thus, WMI has been proposed to be a therapeutic target for primary and secondary prevention of cognitive impairment and progression to dementia.^[^ ^6^ ^]^ Recent studies have shown that effective hypertension management can reduce WMH volume;^[^ ^7^ ^]^ however, in some clinical studies, the management of vascular risk factors failed to alleviate WMI progression and cognitive decline,^[^ ^8^ ^]^ suggesting that while current strategies can mitigate or reverse WMI, an effective, stable, and widely applicable therapeutic approach for WMI is still needed.

Ischemic WMI reflects demyelination resulting from cerebral artery stenosis or occlusion.^[^ ^9^ ^]^ Myelin sheaths that enwrap axons accelerate the propagation of action potentials, which is the biological basis of signal transduction in neurons.^[^ ^10^ ^]^ Moreover, neurons have been reported to promote oligodendrocyte differentiation or myelin generation in an activity‐dependent manner, a process that is now widely called adaptive myelination.^[^ ^11^ ^]^ A recent study demonstrated that optogenetic activation of the premotor cortex can promote oligodendrogenesis and improve myelination in the corpus callosum.^[^ ^12^ ^]^ Optogenetics and chemogenetics have important neuronal modulation effects. In contrast to traditional electrical stimulation, both methods allow manipulation of specific types of neurons with a high spatiotemporal resolution in freely moving animals.^[^ ^13^ ^]^ Specifically, optogenetics enables direct control of target neurons by using light‐sensitive channel proteins. Specific neurons expressing these channels can be activated by the light source in milliseconds.^[^ ^14^ ^]^ However, since long‐lasting neuronal activation is required to achieve adaptive myelination while optical activation is transient, CNO‐based chemogenetics, which allows long‐lasting neuronal activation (≈10 h), provides an alternative strategy for investigating adaptive myelination. Additionally, repetitive transcranial magnetic stimulation (rTMS) is a noninvasive technique to modulate neuronal activity in the superficial cortex and has been widely used in the treatment of several neuropsychological diseases (e.g., depression).^[^ ^15^ ^]^ The results of optogenetic studies might provide the basis for the clinical transformation of neuroregulatory therapy for directing myelin repair and improving cognitive function in patients with WMH.

In this study, we used bilateral carotid artery stenosis (BCAS) to induce WMHs of vascular origin related to cognitive decline. The BCAS model exhibited working memory deficits due to myelin loss in the corpus callosum, caudate putamen, and internal capsule, which is consistent with the pathological and clinical findings in human patients with WMH of vascular origin.^[^ ^16^ ^]^ Furthermore, we found that projection fibers from glutamatergic neurons in the medial prefrontal cortex (mPFC) passed through the corpus callosum. Optogenetic and chemogenetic activation of mPFC glutamatergic neurons after BCAS attenuated cognitive impairment, facilitated OPC differentiation, and promoted remyelination in the corpus callosum. Mechanistically, neuronal activation upregulated Wnt2 expression, which contributed to the remyelination‐promoting effects of neuromodulation. Collectively, our data demonstrated that mPFC activation reduces myelin loss and alleviates cognitive impairment in the context of ischemic WMI, highlighting the transformative and therapeutic potential of neuromodulation in attenuating WMI‐related cognitive decline.

## 2 Results

### 2.1 mPFC Neuronal Stimulation Alleviates WMI‐Related Cognitive Impairment

**Figure 1:**
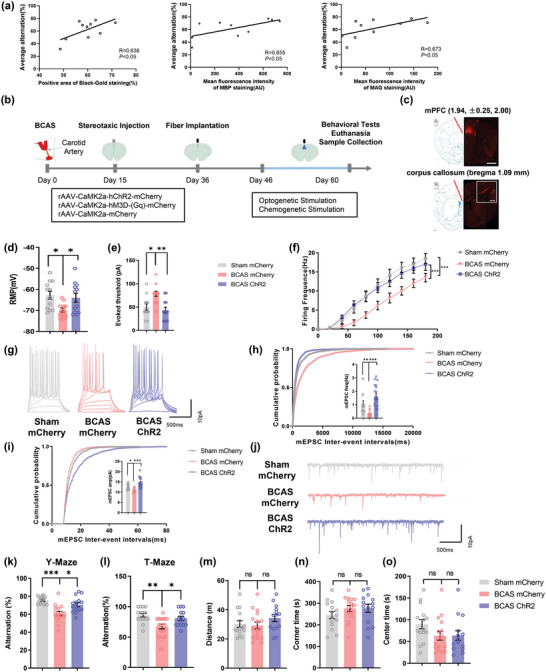
Optogenetic stimulation alleviates WMI‐related cognitive impairment in mice. a) Correlation between white matter injury and cognitive decline 2 months after surgery in BCAS model mice. White matter injury was evaluated by Black‐gold staining and immunofluorescence staining. Cognitive function was evaluated by the T‐maze test and Y‐maze test. The mean spontaneous alternation percentage in the T‐maze and Y‐maze tests was used as an index of cognitive function. *n* = 10. b) Schematic diagram of stereotaxic injection into BCAS model mice followed by long‐term optogenetic and chemogenetic stimulation. c) rAAV‐CaMKII*α*‐mCherry‐WPRE‐hGH pA was injected into the medial prefrontal cortex (mPFC) and was transported to the corpus callosum. Representative confocal images of mCherry expression in the mPFC and corpus callosum. Scale bar: 1 mm (top); 400 µm (bottom). d) The resting membrane potential (RMP), e) action potential (AP) threshold in response to current injection, and f) firing frequency of pyramidal neurons in layer 2/3 of the mPFC in the sham mCherry, BCAS mCherry, and BCAS ChR2 groups at 2 months after surgery. Recordings were acquired from 3 mice per group. *n* = 12 recordings per group. g) Representative APs of pyramidal neurons in layers 2/3 of the mPFC in the sham mCherry, BCAS mCherry, and BCAS ChR2 groups at 2 months after surgery. The h) frequency and i) amplitude of miniature excitatory postsynaptic currents (mEPSCs) of glutamatergic neurons in the mPFC in the sham mCherry, BCAS mCherry, and BCAS ChR2 groups at 2 months after surgery. Recordings were acquired from 3 mice per group. *n* = 16–18 recordings per group. j) Representative mEPSCs of glutamatergic neurons in the mPFC in the sham mCherry, BCAS mCherry, and BCAS ChR2 groups at 2 months after surgery. Results of k) Y‐maze and l) T‐maze tests showing the spontaneous alternation percentage, defined as the proportion of times that a mouse entered all three arms consequently and the proportion of times a mouse entered the correct goal arm, in the sham mCherry, BCAS mCherry, and BCAS ChR2 groups at 2 months after surgery. *n* = 15–16 per group. Results of the open field test showing m) the total distance travelled and n) time spent in the corner area and o) center area in the sham mCherry, BCAS mCherry, and BCAS ChR2 groups at 2 months after surgery. *n* = 15–16 per group. The data are presented as the mean ± SEM. *p*‐values were determined by Pearson's rank correlation in (a); by the Kruskal–Wallis test with Dunn's post‐hoc analysis in (e), (h), (k), (m), and (o); by 1‐way ANOVA with Tukey's post‐hoc analysis in (d), (i), (l), and (n); and by 2‐way ANOVA with Tukey's post‐hoc analysis in (f). *p < 0.05, **p < 0.01, ***p < 0.001.

To explore the correlation between WMI and cognitive impairment, we used the BCAS model, which exhibits WMH‐related cognitive decline. Black‐gold staining and immunofluorescence staining of myelin basic protein (MBP) and myelin‐associated glycoprotein (MAG) were used to evaluate WMI. Cognitive decline was assessed by the Y‐maze and T‐maze tests. The results demonstrated that WMI was positively associated with cognitive decline (**Figure** **1**a) (black‐gold: *R* = 0.638, *p <* 0.05; MBP: *R* = 0.655, *p <* 0.05; MAG: *R* = 0.673, *p <* 0.05; *n* = 10), indicating the occurrence of WMI‐mediated cognitive dysfunction.

The corpus callosum is the largest white matter tract in the brain and is susceptible to ischemic injury. We aimed to alleviate WMI through adaptive myelination by targeting the mPFC, which projects through the corpus callosum, to investigate whether neuromodulation of the mPFC can ameliorate WMI. According to the experimental schedule presented in Figure 1b, ChR2‐mCherry or a control mCherry adeno‐associated virus (AAV) was injected into the bilateral mPFC PrL layers 2/3 for anterograde transport through callosal projections (Figure 1c), which revealed efficient and selective transduction in glutamatergic neurons (Figure S1a–c, Supporting Information), and glutamatergic neurons were then repeatedly activated by photostimulation at low frequency (20 Hz) for 14 consecutive days starting from 46 days post‐BCAS.

Then, we employed electrophysiological techniques to confirm the activation of glutamatergic neurons in layers 2/3 of the mPFC after optogenetic stimulation. The results indicated that the resting membrane potential (RMP) and the firing frequency of mPFC glutamatergic neurons were decreased in the BCAS group (RMP: BCAS mCherry group versus Sham mCherry group, *p* < 0.05; firing frequency: BCAS mCherry group versus Sham mCherry group, *p* < 0.001), while the threshold current showed the opposite pattern in the BCAS group (BCAS mCherry group vs Sham mCherry group, *p* < 0.05); this effect was reversed by optogenetic stimulation (RMP: BCAS ChR2 group vs BCAS mCherry group, *p* < 0.05; firing frequency: BCAS ChR2 group vs BCAS mCherry group, *p* < 0.001; threshold current: BCAS ChR2 group vs BCAS mCherry group, *p* < 0.01) (Figure 1d–g). We assessed the excitatory synaptic function of mPFC glutamatergic neurons and found that the frequency and amplitude of miniature excitatory postsynaptic currents (mEPSCs) were decreased in the BCAS group and that optogenetic activation effectively reversed this decline in mEPSC frequency and amplitude (mEPSC‐Freq: BCAS mCherry group vs Sham mCherry group, *p* < 0.01; BCAS ChR2 group vs BCAS mCherry group, *p* < 0.001; mEPSC‐Amp: BCAS mCherry group vs Sham mCherry group, *p* < 0.05; BCAS ChR2 group vs BCAS mCherry group, *p* < 0.001; *n* = 3 per group) (Figure 1h–j).

After that, behavioral tests were applied to evaluate the cognitive function of the mice. The results indicated that the mice showed a lower percentage of spontaneous alterations in the Y‐maze and T‐maze tests at 2 months post‐BCAS (Y‐maze: BCAS mCherry group vs Sham mCherry group, *p* < 0.001; T‐maze test: BCAS mCherry group vs Sham mCherry group *p* < 0.01). However, optogenetic stimulation reversed these behavioral changes (Y‐maze: BCAS ChR2 group vs BCAS mCherry group, *p <* 0.05; T‐maze test: BCAS ChR2 group vs BCAS mCherry group, *p <* 0.05; *n* = 15–16 per group) (Figure 1k,l), suggesting that the optogenetic activation of glutamatergic neurons in the mPFC ameliorated short‐term working memory impairment. In addition, we also examined locomotor activity and anxiety‐like behavior, and the open field test showed no significant difference in the distance travelled or the time spent in the corner and center area (Figure 1m–o).

**Figure 2:**
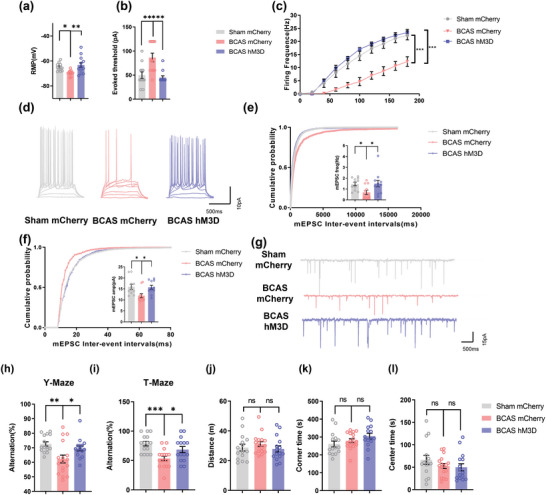
Chemogenetic stimulation ameliorates WMI‐related cognitive impairment in mice. a) The resting membrane potential (RMP), b) action potential (AP) threshold in response to current injection, and c) firing frequency of pyramidal neurons in layer 2/3 of the mPFC in the sham mCherry, BCAS mCherry, and BCAS hM3D groups at 2 months after surgery. Recordings were acquired from 3 mice per group. *n* = 12 recordings per group. d) Representative APs of pyramidal neurons in layers 2/3 of the mPFC in the sham mCherry, BCAS mCherry, and BCAS hM3D groups at 2 months after surgery. e) The frequency and f) amplitude of miniature excitatory postsynaptic currents (mEPSCs) of glutamatergic neurons in the mPFC in the sham mCherry, BCAS mCherry, and BCAS hM3D groups at 2 months after surgery. Recordings were acquired from 3 mice per group. *n* = 11 recordings per group. g) Representative mEPSCs of glutamatergic neurons in the mPFC in the sham mCherry, BCAS mCherry, and BCAS hM3D groups at 2 months after surgery. Results of h) Y‐maze and i) T‐maze tests showing the spontaneous alternation percentage of the sham mCherry, BCAS mCherry, and BCAS hM3D groups at 2 months after surgery. *n* = 15–17 per group. Results of the open field test showing j) the total distance travelled and k) time spent in the corner area and l) center area in the sham mCherry, BCAS mCherry, and BCAS hM3D groups at 2 months after surgery. *n* = 15–17 per group. The data are presented as the mean ± SEM. *p*‐values were determined by 1‐way ANOVA with Tukey's post‐hoc analysis in (a), (e), (h), (i), and (k); by the Kruskal–Wallis test with Dunn's post‐hoc analysis in (b), (f), (j), and (l); and by 2‐way ANOVA with Tukey's post‐hoc analysis in (c). *p < 0.05, **p < 0.01, ***p < 0.001.

Furthermore, we used a chemogenetic approach to validate the above results. Notably, as in optogenetic experiments, the in vitro electrophysiology results demonstrated that chemogenetic stimulation could rescue the decrease in the excitability and synaptic function of pyramidal neurons in the mPFC of BCAS model mice (**Figure** **2**a–g). Accordingly, chemogenetic activation of the mPFC also improved the working memory of BCAS model mice (Figure 2h–l). Collectively, these results showed that WMI‐related memory deficits in mice can be alleviated by the activation of glutamatergic neurons in the mPFC.

### 2.2 Activation of Glutamatergic Neurons in the mPFC Leads to White Matter Repair

**Figure 3:**
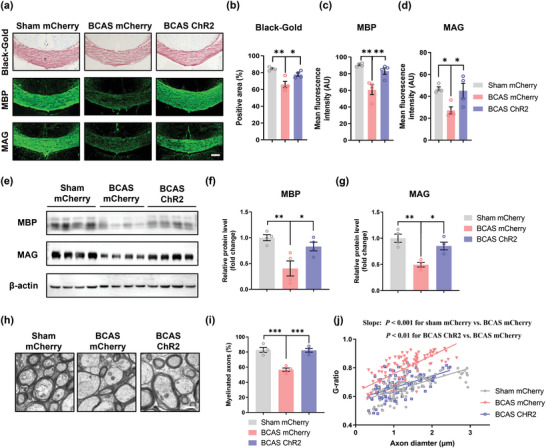
Optogenetic stimulation promotes myelin repair in mice. a) Representative images of Black‐gold staining and immunostaining of MBP and MAG in the corpus callosum in the sham mCherry, BCAS mCherry, and BCAS ChR2 groups at 2 months after surgery. Scale bar: 200 µm. Quantification of b) Black‐gold staining and immunostaining of c) MBP and d) MAG in the corpus callosum in the sham mCherry, BCAS mCherry, and BCAS ChR2 groups at 2 months after surgery. *n* = 4 per group. e) Immunoblot bands and quantification of f) MBP and g) MAG expression in the corpus callosum in the sham mCherry, BCAS mCherry, and BCAS ChR2 groups at 2 months after surgery. The intensity of each immunoblot band was normalized to that of the *β*‐actin band. The measured values were normalized to the mean value of the sham group. *n* = 4 per group. h) Representative TEM images of the corpus callosum in the sham mCherry, BCAS mCherry, and BCAS ChR2 groups at 2 months after surgery. Scale bar: 1 µm. i) The percentage of myelinated axons in the corpus callosum in the sham mCherry, BCAS mCherry, and BCAS ChR2 groups at 2 months after surgery. *n* = 4 per group. j) Scatterplots of the myelin g‐ratio as a function of the axon diameter in the corpus callosum in the sham mCherry, BCAS mCherry, and BCAS ChR2 groups at 2 months after surgery. Axons were selected from 4 mice per group for measurement. *n* = 114, *n* = 115, and *n* = 105 measured axons for each group. The data are presented as the mean ± SEM. *p*‐values were determined by 1‐way ANOVA with Tukey's post‐hoc analysis in (b), (c), (d), (f), (g) and (i) and by the Kruskal–Wallis test with Dunn's post‐hoc analysis in (j). *p < 0.05, **p < 0.01, ***p < 0.001.

Since adaptive myelination is defined as myelin plasticity regulated by neuronal activity, we next wondered whether mPFC activation facilitates myelin repair, which then contributes to improvements in cognition in BCAS model mice. First, we assessed the pathological changes in the corpus callosum. Black‐gold staining revealed severe demyelination in the corpus callosum in BCAS model mice, while mice in which the mPFC was optogenetically activated showed preservation of white matter integrity (BCAS mCherry group vs Sham mCherry group, *p* < 0.01; BCAS ChR2 group vs BCAS mCherry group, *p* < 0.05). Then, immunofluorescence staining and western blotting were used to measure the expression levels of MBP and MAG in the corpus callosum. MBP and MAG expression was downregulated in BCAS model mice compared with sham mice and increased by photostimulation (**Figure** **3**a–d: MBP: BCAS mCherry group vs Sham mCherry group, *p* < 0.01; BCAS ChR2 group vs BCAS mCherry group, *p* < 0.01; MAG: BCAS mCherry group vs Sham mCherry group, *p <* 0.05; BCAS ChR2 group vs BCAS mCherry group, *p* < 0.05; *n* = 4 per group) (Figure 3a–g). Moreover, transmission electron microscopy (TEM) was applied to observe the submicroscopic structural changes in the corpus callosum. TEM revealed that the percentage of myelinated axons was significantly decreased after BCAS and that optogenetic activation increased the percentage of myelinated axons in the corpus callosum in BCAS model mice (BCAS mCherry group vs Sham mCherry group, *p* < 0.001; BCAS ChR2 group vs BCAS mCherry group, *p* < 0.001) (Figure 3h,i). We also evaluated the myelination of axons in the corpus callosum by calculating the g‐ratio (the ratio of the axonal diameter to the myelin sheath thickness). The results revealed a significant increase in the g‐ratio in BCAS model mice and a decrease in the g‐ratio after stimulation (BCAS mCherry group vs Sham mCherry group, *p* < 0.001; BCAS ChR2 group vs BCAS mCherry group, *p* < 0.01; *n* = 4 per group) (Figure 3j).

**Figure 4:**
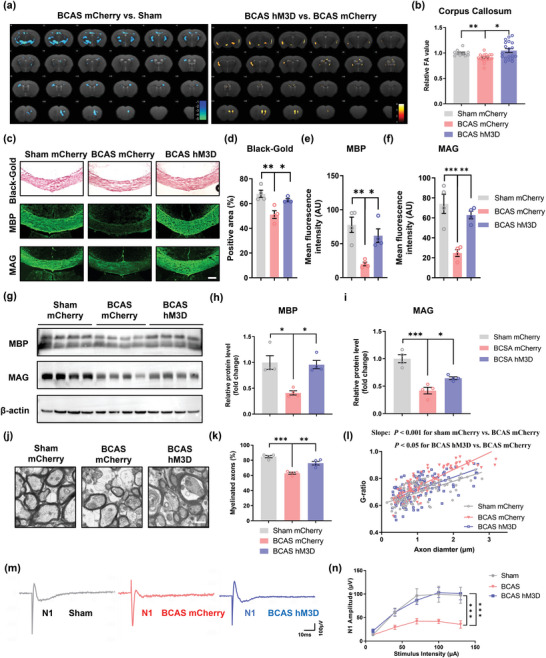
Chemogenetic stimulation promotes myelin repair in mice. a) Heatmaps generated from DTI axial views of FA acquired from sham mCherry, BCAS mCherry, and BCAS hM3D groups at 2 months after surgery. b) Quantification of FA values in the corpus callosum in the sham mCherry, BCAS mCherry, and BCAS hM3D groups at 2 months after surgery. The measured values were normalized to the mean value of the sham group. *n* = 11 in the sham mCherry group, *n* = 20 in the BCAS mCherry group and *n* = 19 in the BCAS hM3D group. c) Representative images of Black‐gold staining and immunostaining of MBP and MAG in the corpus callosum in the sham mCherry, BCAS mCherry, and BCAS hM3D groups at 2 months after surgery. Scale bar: 200 µm. Quantification of d) Black‐gold staining and immunostaining of e) MBP and f) MAG in the corpus callosum in the sham mCherry, BCAS mCherry, and BCAS hM3D groups at 2 months after surgery. *n* = 4 per group. g) Immunoblot bands and quantification of h) MBP and i) MAG expression in the corpus callosum in the sham mCherry, BCAS mCherry, and BCAS hM3D groups at 2 months after surgery. The intensity of each immunoblot band was normalized to that of the *β*‐actin band. The measured values were normalized to the mean value of the sham group. *n* = 4 per group. j) Representative TEM images of the corpus callosum in the sham mCherry, BCAS mCherry, and BCAS hM3D groups at 2 months after surgery. Scale bar: 1 µm. k) The percentage of myelinated axons in the corpus callosum in the sham mCherry, BCAS mCherry, and BCAS hM3D groups at 2 months after surgery. *n* = 4 per group. l) Scatterplots of the myelin g‐ratio as a function of the axon diameter in the corpus callosum in the sham mCherry, BCAS mCherry, and BCAS hM3D groups at 2 months after surgery. Axons were selected from 4 mice per group for measurement. *n* = 104, *n* = 110, and *n* = 117 measured axons for each group. m) Representative curves of CAPs of myelinated N1 fibers in the corpus callosum in the sham mCherry, BCAS mCherry, and BCAS hM3D groups at 2 months after surgery. n) Quantification of the amplitude of evoked CAPs of myelinated N1 fibers in the corpus callosum in the sham mCherry, BCAS mCherry, and BCAS hM3D groups at 2 months after surgery. *n* = 3 mice and 14–17 recordings per group. The data are presented as the mean ± SEM. *p*‐values were determined by the Kruskal–Wallis test with Dunn's post‐hoc analysis in (b) and (l); by 1‐way ANOVA with Tukey's post‐hoc analysis in (d), (e), (f), (h), (i), and (k); and by 2‐way ANOVA with Tukey's post‐hoc analysis in (n). *p < 0.05, **p < 0.01, ***p < 0.001.

The chemogenetic approach produced similar results as the optogenetic approach (**Figure** **4**a–l). Additionally, as chemogenetic manipulation does not require implantation of optical fibers in the skull, MRI/DTI could be used to assess white matter integrity. Fractional anisotropy (FA) was used to evaluate the microstructure of the white matter.^[^ ^17^ ^]^ The FA value was decreased in BCAS model mice compared to sham mice but increased after chemogenetic activation (BCAS mCherry group vs Sham mCherry group, *p* < 0.01; BCAS hM3D group vs BCAS mCherry group, *p* < 0.05; sham group: *n* = 11, BCAS group: *n* = 20, BCAS hM3D group: *n* = 19).

To test whether remyelination of white matter tracts improves the functional integrity of the white matter, we evaluated compound action potentials (CAPs) of the corpus callosum in coronal brain slices from BCAS model mice that underwent chemogenetic stimulation. The early peak of CAPs represented fast conduction along myelinated axons. The results showed that the peak amplitude was markedly decreased in the BCAS group and that this decrease in peak amplitude was significantly rescued by chemogenetic activation (BCAS mCherry group vs Sham mCherry group, *p* < 0.001; BCAS hM3D group vs BCAS mCherry group, *p* < 0.001; *n* = 3 per group) (Figure 4m,n).

### 2.3 Activation of Glutamatergic Neurons in the mPFC Drives Oligodendrocytes Differentiation

**Figure 5:**
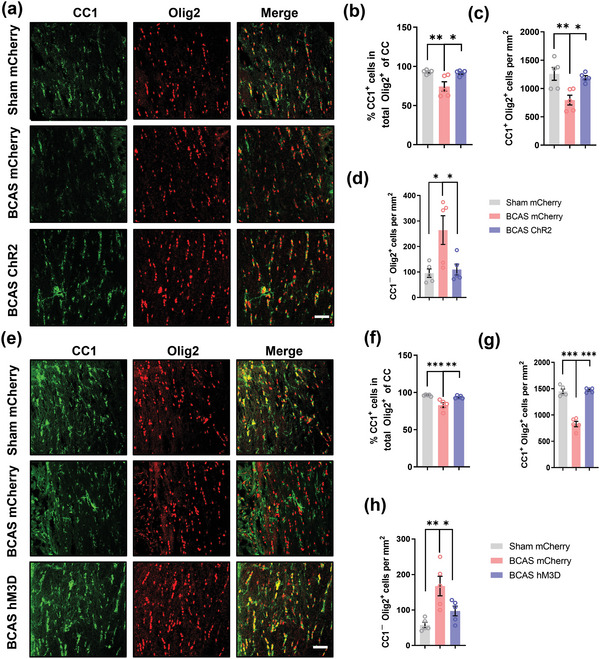
Stimulation of prefrontal cortex neurons drives oligodendrocyte differentiation. a) Representative immunostaining of CC1 and Olig2 in the corpus callosum in the sham mCherry, BCAS mCherry, and BCAS ChR2 groups at 2 months after surgery. Scale bar: 50 µm. b) Proportion of CC1^+^/Olig2^+^ cells in the corpus callosum in the sham mCherry, BCAS mCherry, and BCAS ChR2 groups at 2 months after surgery. *n* = 5 per group. Density of c) CC1^+^Olig2^+^ cells and d) CC1^−^Olig2^+^ cells in the corpus callosum in the sham mCherry, BCAS mCherry, and BCAS ChR2 groups at 2 months after surgery. *n* = 5 per group. e) Representative immunostaining of CC1 and Olig2 in the corpus callosum in the sham mCherry, BCAS mCherry, and BCAS hM3D groups at 2 months after surgery. Scale bar: 50 µm. f) Proportion of CC1^+^/Olig2^+^ cells in the corpus callosum in the sham mCherry, BCAS mCherry, and BCAS hM3D groups at 2 months after surgery. *n* = 5 per group. Density of g) CC1^+^Olig2^+^ cells and h) CC1^−^Olig2^+^ cells in the corpus callosum in the sham mCherry, BCAS mCherry, and BCAS hM3D groups at 2 months after surgery. *n* = 5 per group. The data are presented as the mean ± SEM. *p*‐values were determined by 1‐way ANOVA with Tukey's post‐hoc analysis in (b–d) and (f–h). *p < 0.05, **p < 0.01, ***p < 0.001.

To further investigate whether enhanced remyelination is associated with changes in the oligodendrocyte pool, we performed immunofluorescence staining for Olig2, an oligodendrocyte lineage cell marker, and CC1, a mature oligodendrocyte marker, to distinguish different cell stages in brain sections (**Figure** **5**a). The proportion of CC1^+^/Olig2^+^ cells in the corpus callosum was reduced in BCAS model mice compared with sham mice, while optogenetic activation of the mPFC increased the proportion of these cells (BCAS mCherry group vs Sham mCherry group, *p* < 0.01; BCAS ChR2 group vs BCAS mCherry group, *p <* 0.05; *n* = 5 per group) (Figure 5b). The density of CC1^+^ Olig2^+^ cells also exhibited a similar pattern (BCAS mCherry group vs Sham mCherry group, *p* < 0.01; BCAS ChR2 group vs BCAS mCherry group, *p <* 0.05) (Figure 5c). Moreover, the density of CC1^−^Olig2^+^ cells was elevated in the BCAS group and decreased after photostimulation (BCAS mCherry group vs Sham mCherry group, *p* < 0.05; BCAS ChR2 group vs BCAS mCherry group, *p <* 0.05) (Figure 5d). Additionally, we used Ki67 to label proliferating cells, and the results demonstrated that the density of Ki67^+^Olig2^+^ cells remained unchanged (Figure S2a,b, Supporting Information). To further explore the changes in the oligodendrocyte lineage, we administered EdU during neuromodulation to trace the newly generated cells. Costaining for EdU, Olig2, and CC1 showed that the density of EdU^+^Olig2^+^ cells in the corpus callosum did not change significantly, while the proportion of CC1^+^ cells in total EdU^+^ cells of corpus callosum increased after treatment (Figure S3a–c,f–g, Supporting Information). Consistently, chemogenetic stimulation had a similar effect (Figure 5e–h; Figures S3d,e,h,i and S4, Supporting Information).

Furthermore, TUNEL staining showed little change in the number of dying oligodendrocyte lineage cells among all these groups (Figure S5a–d, Supporting Information). All the results above indicated that neuromodulation of mPFC glutamatergic neurons mainly regulates the differentiation rather than the proliferation or survival of oligodendrocyte lineage cells in the BCAS model.

Although the abovementioned data confirmed that OPC proliferation was not affected by mPFC activation, we found that, compared to the BCAS group, the number of Olig2^+^ cells in the corpus callosum actually increased approximately 400 cells mm^−2^ after neuronal stimulation (Figure S5e,f, Supporting Information). Since the subventricular zone (SVZ) is close to the corpus callosum, the relevant signaling pathway activated by neuronal stimulation might promote the migration of OPCs from the SVZ to the corpus callosum.

### 2.4 Activation of Glutamatergic Neurons in the mPFC Upregulates Wnt2 Expression in the Corpus Callosum

**Figure 6:**
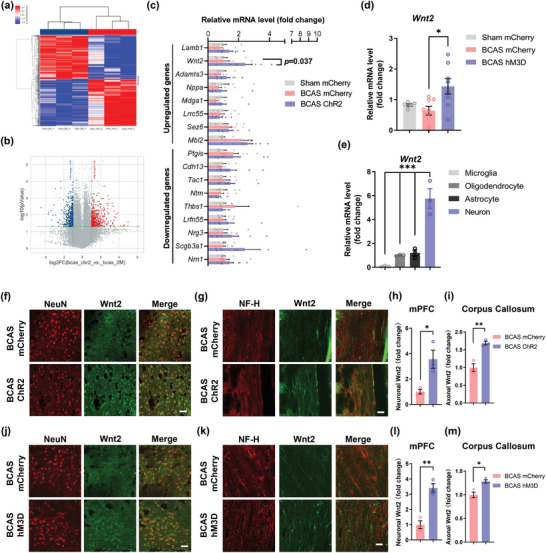
Activation of glutamatergic neurons in the mPFC upregulates Wnt2 expression in the corpus callosum. a) Heatmap of the results of mPFC tissue microarray analysis between the BCAS mCherry and BCAS ChR2 groups at 2 months after surgery. *n* = 3 per group. b) Volcano plot of the results of mPFC tissue microarray analysis between the BCAS mCherry and BCAS ChR2 groups at 2 months after surgery (fold change > 2; *p* < 0.05). The DEGs are listed. *n* = 3 per group. c) Real‐time PCR for validation of the identified DEGs among the sham mCherry, BCAS mCherry, and BCAS ChR2 groups at 2 months after surgery. *n* = 7–8 per group. d) Real‐time PCR analysis of *Wnt2* expression in the mPFC in the sham mCherry, BCAS mCherry, and BCAS hM3D groups at 2 months after surgery. *n* = 7–8 per group. e) Real‐time PCR analysis of *Wnt2* expression in different types of primary cortical neural cells. *n* = 3 per group. f) Representative immunostaining of NeuN and Wnt2 in the mPFC in the sham mCherry, BCAS mCherry, and BCAS ChR2 groups at 2 months after surgery. Scale bar: 50 µm. g) Representative immunostaining of NF‐H and Wnt2 in the corpus callosum in the sham mCherry, BCAS mCherry, and BCAS ChR2 groups at 2 months after surgery. Scale bar: 35 µm. Quantification of immunostaining of Wnt2 in the h) mPFC and i) corpus callosum in the sham mCherry, BCAS mCherry, and BCAS ChR2 groups at 2 months after surgery. The fluorescence intensity in each image was normalized to that of NeuN and NF‐H. The measured values were normalized to the mean value of the BCAS group. *n* = 3 per group. j) Representative immunostaining of NeuN and Wnt2 in the mPFC in the sham mCherry, BCAS mCherry, and BCAS hM3D groups at 2 months after surgery. Scale bar: 50 µm. k) Representative immunostaining of NF‐H and Wnt2 in the corpus callosum in the sham mCherry, BCAS mCherry, and BCAS hM3D groups at 2 months after surgery. Scale bar: 35 µm. Quantification of Wnt2 immunostaining in the l) mPFC and m) corpus callosum in the sham mCherry, BCAS mCherry, and BCAS hM3D groups at 2 months after surgery. The fluorescence intensity in each image was normalized to that of NeuN and NF‐H. The measured values were normalized to the mean value of the BCAS group. *n* = 3 per group. The data are presented as the mean ± SEM. *p*‐values were determined by the Kruskal–Wallis test with Dunn's post‐hoc analysis in (d); by 1‐way ANOVA with Tukey's post‐hoc analysis in (c) and (e); and by Student's *t*‐test in (h), (i), (l), and (m). *p < 0.05, **p < 0.01.

Activated neurons have been reported to secrete various factors that nourish oligodendrocyte lineage cells, promoting their differentiation and myelination. We thus postulated that soluble factors derived from axons of activated neurons might enhance myelin repair in our model. To further test our hypothesis, we performed microarray‐based genomics analysis of mPFC tissues and found 403 upregulated and 373 downregulated genes after optogenetic activation (**Figure** **6**a,b). Next, we searched these differentially expressed genes (DEGs) in the Cell Type Expression Correlates (http://oldhamlab.ctec.ucsf.edu/) and UniProt databases to screen for secreted proteins specifically expressed by neurons. qPCR, which was used to validate the mRNA expression levels of these genes (*Lamb1, Wnt2, Adamts3, Nppa, Mdga1, Lrrc55, Sez6, Mbl2, Ptgis, Cdh13, Tac1, Ntm, Thbs1, Lrfn55, Nrg3, Scgb3a1*, and *Nrn1*), revealed that only *Wnt2* showed 2.3 times higher expression in optogenetically activated mPFCs (Figure 6c). The expression of *Wnt2* was also confirmed to be upregulated in the mPFC after chemogenetic activation (2.24‐fold) (Figure 6d). Furthermore, *Wnt2* mRNA levels were highly expressed in primary cortical neurons compared with other neural cells (Figure 6e). Next, the protein levels of Wnt2 in the mPFC and corpus callosum were examined by immunofluorescence staining. The results confirmed that the protein expression of Wnt2 was correspondingly upregulated after neuronal activation and that the Wnt2 protein was localized along axons (mPFC: BCAS ChR2 group vs BCAS mCherry group, *p* < 0.05; BCAS hM3D group vs BCAS mCherry group, *p* < 0.01; corpus callosum: BCAS ChR2 group vs BCAS mCherry group, *p* < 0.01; BCAS hM3D group vs BCAS mCherry group, *p* < 0.05; *n* = 3 per group) (Figure 6f–m; Figure S6a, Supporting Information). To further validate that Wnt signaling was involved in this process, we also explored the expression of *β*‐catenin, a key component of Wnt signaling. The results revealed that mPFC activation significantly upregulated the expression of *β*‐catenin in Olig2^+^ cells (Figure S6b,c, Supporting Information). Collectively, these data suggested that Wnt2 expression is upregulated in optogenetically and chemogenetically activated mPFC neurons and that these neurons might actively secrete Wnt2 in the corpus callosum to activate Wnt signaling in oligodendrocyte lineage cells in the BCAS model.

### 2.5 Overexpression of *Wnt2* in mPFC Glutamatergic Neurons Alleviates Myelin Injury and Improves Cognition

**Figure 7:**
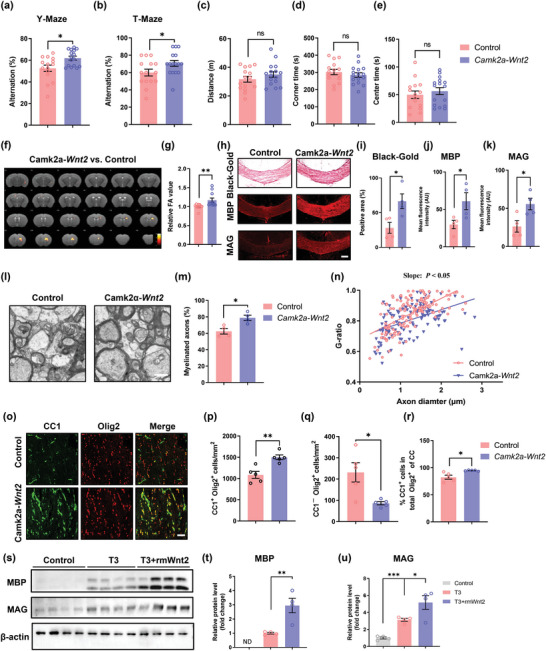
Overexpression of Wnt2 in mPFC glutamatergic neurons alleviates myelin injury and improves cognition. Results of the a) Y‐maze and b) T‐maze tests showing the spontaneous alternation percentage in the control and *Camk2a‐Wnt2* groups at 2 months after surgery. *n* = 15–16 per group. Results of the open field test showing c) the total distance travelled and d) time spent in the corner area and e) center area in the control and *Camk2a‐Wnt2* groups at 2 months after surgery. *n* = 15–16 per group. f) Heatmaps generated from DTI axial views of FA acquired from the control and *Camk2a‐Wnt2* groups at 2 months after surgery. g) Quantification of FA values in the corpus callosum in the control and *Camk2a‐Wnt2* groups at 2 months after surgery. The measured values were normalized to the mean value of the control group. *n* = 11 per group. h) Representative images of Black‐gold staining and immunostaining of MBP and MAG in the corpus callosum in the control and *Camk2a‐Wnt2* groups at 2 months after surgery. Scale bar: 200 µm. Quantification of i) Black‐gold staining and immunostaining of j) MBP and k) MAG in the corpus callosum in the control and *Camk2a‐Wnt2* groups at 2 months after surgery. *n* = 4 per group. l) Representative TEM images of the corpus callosum in the control and *Camk2a‐Wnt2* groups at 2 months after surgery. Scale bar: 1 µm. m) The percentage of myelinated axons in the corpus callosum in the control and *Camk2a‐Wnt2* groups at 2 months after surgery. *n* = 4 per group. n) Scatterplots of the myelin g‐ratio as a function of the axon diameter in the corpus callosum in the control and *Camk2a‐Wnt2* groups at 2 months after surgery. Axons were selected from 4 mice per group for measurement. *n* = 138 and *n* = 121 measured axons for each group, respectively. o) Representative immunostaining of CC1 and Olig2 in the corpus callosum in the control and *Camk2a‐Wnt2* groups at 2 months after surgery. Scale bar: 50 µm. Density of p) CC1^+^Olig2^+^ cells and q) CC1^−^Olig2^+^ cells in the corpus callosum in the control and *Camk2a‐Wnt2* groups at 2 months after surgery. *n* = 5 per group. r) Proportion of CC1^+^/Olig2^+^ cells in the corpus callosum in the control and *Camk2a‐Wnt2* groups at 2 months after surgery. *n* = 5 per group. s) Immunoblot bands and t) quantification of MBP and u) MAG expression in OPCs in the control, T3, and T3+ rmWnt2 groups. The intensity of each immunoblot band was normalized to that of the *β*‐actin band. The measured values were normalized to the mean value of the control group. *n* = 3 per group. The data are presented as the mean ± SEM. *p*‐values were determined by Student's *t*‐test in (b–e), (i–k), (m), (p–r), and (t); by the Mann–Whitney test in (a), (g), and (n); and by 1‐way ANOVA with Tukey's post‐hoc analysis in (u). *p < 0.05, **p < 0.01, ***p < 0.001.

To verify whether mPFC neuron‐derived Wnt2 is involved in remyelination, we overexpressed *Wnt2* in the mPFC via stereotactic injection of an AAV expressing exogenous *Wnt2*. First, we assessed the AAV transduction efficiency. The mRNA levels of *Wnt2* in the mPFC were increased by approximately 197‐fold in the *Wnt2* overexpression group compared to the control group (Figure S7a, Supporting Information). Behavioral tests were performed 67 days after virus injection. The results indicated that *Wnt2* overexpression improved the cognitive function of mice (Y maze: *Camk2a‐Wnt2* group vs control group, *p* < 0.05; T maze: *Camk2a‐Wnt2* group vs control group, *p* < 0.05; *n* = 15–16 per group) (**Figure** **7**a–e). MRI/DTI also showed that the FA value was higher in *Wnt2*‐overexpressing mice (*Camk2a‐Wnt2* group vs control group, *p* < 0.01; *n* = 11 per group) (Figure 7f,g). To further explore the effect of *Wnt2* overexpression on myelin changes in the corpus callosum (Figure 7h–k), black‐gold staining (*Camk2a‐Wnt2* group vs control group, *p* < 0.05) and immunofluorescence staining (MBP: *Camk2a‐Wnt2* group vs control group, *p* < 0.05; MAG: *Camk2a‐Wnt2* group vs control group, *p* < 0.05; *n* = 4 per group) were subsequently performed. The results showed that myelin integrity was restored in mice overexpressing *Wnt2*. The results of western blotting also demonstrated that  the expression of MAG and MBP was upregulated after *Wnt2* overexpression (Figure S7b–d, Supporting Information). TEM revealed an increase in the percentage of myelinated axons (*Camk2a‐Wnt2* group vs control group, *p* < 0.01) and a lower g‐ratio (*Camk2a‐Wnt2* group vs control group, *p* < 0.05; *n* = 5 per group) in *Wnt2*‐overexpressing mice (Figure 7l–n). Regarding the effect of Wnt2 on oligodendrocytes, we observed that the proportion of CC1^+^/Olig2^+^ cells (*Camk2a‐Wnt2* group vs control group, *p* < 0.05) and the density of CC1^+^Olig2^+^ cells (*Camk2a‐Wnt2* group vs control group, *p* < 0.05; *n* = 5 per group) were both increased after *Wnt2* overexpression (Figure 7o–r).

To further explore whether Wnt2 directly promotes the differentiation of OPCs, we treated cultured OPCs with recombinant mouse Wnt2 (rmWnt2) and found that it promoted the triiodothyronine (T3)‐induced differentiation of OPCs (Figure 7s,u). Next, to determine the underlying mechanism of Wnt2‐potentiated OPC differentiation, we treated cultured OPCs with recombinant Wnt2 and Dkk1, an antagonist of Wnt signaling. The results showed that Dkk1 significantly attenuated the effects of rmWnt2 (Figure S7e–g, Supporting Information). Therefore, our data validated that neuronal Wnt2 drove oligodendrocyte differentiation, promoted myelin repair, and ameliorated cognitive impairment in BCAS model mice via the canonical Wnt/*β*‐catenin signaling pathway, possibly contributing to the beneficial effect of mPFC activation on white matter integrity.

## 3 Discussion

White matter consists mostly of myelinated axons, and constant damage to myelinated axons can lead to progressive cognitive decline.^[^ ^18^ ^]^ Neuroimaging studies by our group and others previously found that structural and functional dysconnectivity contributes to WMI‐related executive dysfunction.^[^ ^19^ ^]^ As the largest white matter tract in the brain, the corpus callosum connects the two hemispheres and acts as an integrative hub that maintains executive function, including working memory, as patients with agenesis of the corpus callosum can develop executive deficits.^[^ ^20^ ^]^ More importantly, loss of corpus callosum integrity has been shown to be associated with cognitive decline, especially impaired executive processing, in subjects with WMI.^[^ ^21^ ^]^ The BCAS rodent model is commonly used to mimic cognitive deficits in humans caused by cerebral hypoperfusion due to vascular diseases.^[^ ^16^ ^]^ In this study, we found that the extent of working memory impairment was positively correlated with that of myelin loss in the corpus callosum, indicating that slowing or reversing WMI might be a promising therapeutic strategy for WMI‐related cognitive deficits. However, due to the lack of pharmacological targets with promyelination potential and the controversial findings related to the effects of vascular risk management, novel approaches for ameliorating WMI are needed.

In our study, anterograde tracing with AAV‐mCherry confirmed that projection fibers from glutamatergic neurons in the mPFC PrL layers 2/3 passed through the corpus callosum, which was supported by previous findings.^[^ ^22^ ^]^ In addition, worsening of functional connectivity between the PFC and subcortical nucleus is closely linked with disruption of white matter integrity and a decline in cognitive performance.^[^ ^23^ ^]^ Moreover, pyramidal neurons in layers 2/3 are the most abundant cells in the neocortex.^[^ ^24^ ^]^ We thus chose mPFC neurons as targets for neuromodulation. Consistent with the concept of adaptive myelination, we found that both optogenetic and chemogenetic activation of mPFC neurons facilitated remyelination in the corpus callosum, restored callosal conduction velocity, and improved working memory after BCAS.

Oligodendrocyte lineage cells are critical in the process of myelination and remyelination.^[^ ^25^ ^]^ During development and under physiological conditions, OPCs proliferate and migrate to their destinations, where they terminally differentiate into mature oligodendrocytes and generate myelin.^[^ ^26^ ^]^ Adaptive myelination is now recognized as a dynamic process in which the fate of the oligodendrocyte lineage is regulated by neuronal activity.^[^ ^11^ ^]^ For example, sensory or social input, motor experience, and spatial learning have been shown to promote the proliferation and differentiation of OPCs, leading to increased myelination.^[^ ^27^ ^]^ It has also been reported that methotrexate chemotherapy decreases cortical BDNF levels, thus suppressing the proliferation of OPCs and impairing adaptive myelination.^[^ ^28^ ^]^ Furthermore, in a lysophosphatidylcholine‐induced focal demyelination model, repeated optogenetic activation of demyelinated axons promotes oligodendrocyte differentiation and remyelination.^[^ ^29^ ^]^

Thus, we hypothesized that manipulating the activation of neurons using optogenetic and chemogenetic approaches promotes myelin repair and relieves WMI‐related cognitive decline by altering the progression of oligodendrocyte lineages. We found that the number of mature oligodendrocytes and the proportion of mature oligodendrocytes relative to total oligodendrocyte lineage cells were significantly reduced in the BCAS model, which is consistent with previous studies using the same rodent model,^[^ ^30^ ^]^ indicating that ischemia‐induced demyelination partly contributes to OPC dysdifferentiation. It has also been reported that the number of OPCs increases after BCAS, suggesting that OPCs respond to chronic ischemia and become proliferatively active, while their failure to differentiate ultimately impair remyelination.^[^ ^31^ ^]^ Our study showed that mPFC activation increased the number of mature oligodendrocytes and decreased the number of OPCs in the BCAS model, while the number of proliferating or dying cells remained unchanged, indicating that neuronal activation mainly regulates the differentiation but not the proliferation nor the survival of oligodendrocyte lineage cells. However, inconsistent with our results, several studies have found that OPCs significantly proliferate after neuronal activation.^[^ ^32^ ^]^ We speculated that because BCAS significantly induced OPC proliferation, the maximum number of OPCs was reached, making them unresponsive to increased neuronal activity. In addition, the effect of neuromodulation on OPC number might vary under physiological and pathological conditions.

The prevalent view of the mechanisms underlying adaptive myelination is that activated neurons change their microenvironmental cues to induce myelination. Many neuronal proteins, including Nrg1, jagged1, and Lingo1, regulate neuron‐oligodendrocyte interactions at various stages of myelination.^[^ ^33^ ^]^ It has also been reported that activated neurons released dynorphin, a neuropeptide, to promote OPC differentiation and the myelination of neighboring axons.^[^ ^34^ ^]^ Since neuron‐derived secreted proteins are capable of driving the differentiation of OPCs toward mature oligodendrocytes and thus increasing myelination, we screened for differentially expressed genes (DEGs) in mPFC neurons in the BCAS model after optogenetic activation by microarray‐based genomics analysis. We found that *Wnt2* expression was significantly upregulated and that *Wnt2* was distributed along the axons of activated neurons, which is similar to previous findings that Wnt3a is enriched in and secreted by neuronal axons.^[^ ^35^ ^]^ A study found that forskolin and KCl induce the depolarization of hippocampal neurons and then trigger the transcription of *Wnt2* in a CREB‐dependent manner, which also supports our finding.^[^ ^36^ ^]^ As expected, overexpression of *Wnt2* in mPFC glutamatergic neurons phenocopied chemogenetic and optogenetic activation‐induced myelin repair after BCAS. Treatment of cultured OPCs with recombinant Wnt2 also potentiated triiodothyronine (T3)‐mediated differentiation, while Dkk1 significantly weakened this effect, indicating that neuron‐derived Wnt2 directly promoted the differentiation of OPCs via the Wnt/*β*‐catenin pathway, which might partly contribute to the therapeutic effects of neuromodulation.

Nevertheless, the role of Wnt signaling in the development of oligodendrocyte lineage cells is still controversial. During the early developmental stage, loss of *β*‐catenin function increased OPC generation from neural stem cells (NSCs).^[^ ^37^ ^]^ In agreement with this, CA‐*β*‐catenin activation or Axin2 mutation led to a decreased number of OPCs.^[^ ^38^ ^]^ In this study, we indicated that Wnt signaling plays a vital role in adaptive myelination after BCAS insult in adult mice. It has been reported that the differentiation of OPCs was delayed in Olig2‐Cre; CA‐*β*‐catenin mutation mice but could return to normal in adults.^[^ ^38^ ^]^ Additionally, the deletion of the Wnt negative regulators Apc and Axin2 inhibited the differentiation of OPCs.^[^ ^39^ ^]^ However, consistent with our findings, Wnt1 treatment could promote OPC differentiation and drive the expression of myelin genes.^[^ ^40^ ^]^ Thus, it has been proposed that the inactivation of Wnt signaling promotes the development of the oligodendrocyte lineage during early CNS development, while its activation is required for the maturation and myelination of oligodendrocytes in adults.^[^ ^41^ ^]^ Collectively, the regulation of OPC development by Wnt signaling might be stage specific, which still needs further investigation with temporal genetic manipulation.

In addition, we observed an increased number of Olig2^+^ cells after neuromodulation. Since the subventricular zone (SVZ) is close to the corpus callosum, and activated Wnt signaling was reported to significantly promote the migration of OPC,^[^ ^42^ ^]^ we speculated that the increased Olig2^+^ cells might be due to increased migration of OPC, which needs further investigation.

However, our current findings have been obtained from young mice, and the therapeutic effect of neuronal modulation in elderly animals remains to be further investigated. These results have important value for clinical conversion therapy on Alzheimer's disease or vascular dementia.

Collectively, our results showed that both optogenetic and chemogenetic activation of mPFC glutamatergic neurons promoted oligodendrocyte differentiation and remyelination and improved working memory in mice after ischemic WMI. Mechanistically, we discovered that activated neuron‐derived Wnt2 is a key molecule linking neuromodulation and adaptive myelination. Our study is the first to introduce the concept of adaptive myelination to the field of myelin repair after ischemic WMI, highlighting its transformative potential in the treatment of WMH‐related cognitive decline.

## 4 Experimental Section

### Animals

Eight‐week‐old male C57/BL6J mice weighing 20–22 grams were purchased from the Model Animal Research Center of Nanjing University (Nanjing, Jiangsu, China). The animals were housed in a room at a controlled temperature on a 12:12 h light:dark cycle and provided free access to standard food and water. All animal experiments were performed according to institutional guidelines and approved by the Animal Care and Use Committee of Nanjing University (reference number: 2019AE01070).

### Bilateral Carotid Artery Stenosis Model

The BCAS model was established as previously reported.^[^ ^16^ ^]^ Briefly, mice were anaesthetized with 2% isoflurane in 33% oxygen, and their body temperature was maintained at 37.0 ± 0.5 °C. The common carotid arteries (CCAs) were exposed from the sheath through a midline cervical incision. The right CCA was carefully lifted and placed between the loops of a microcoil (inner diameter of 0.18 mm, pitch of 0.50 mm, total length of 2.5 mm, Sawane Spring Co., Japan). The microcoil was then twisted around the CCA. The same procedure was performed for the left CCA. Sham‐operated mice underwent all procedures except for microcoil implantation.

### Stereotaxic Intracranial Injection

The mice were anaesthetized with 3% isoflurane in 33% oxygen and then placed in a stereotaxic frame (RWD, China). After the skull was exposed, a thin drill was used to make a small hole in the skull over the medial prefrontal cortex (mPFC) (coordinates: bregma 1.94 mm, lateral ±0.25 mm, depth 2.00 mm). Glass microelectrodes connected to a 10 µL Hamilton microsyringe (micro 4, WPI) were used to inject virus at a depth of 2 mm from bregma over a 10 min period. To avoid outflow of the virus, the microelectrodes were kept in place for 10 min after infusion. For optogenetic stimulation, 200 nL of rAAV‐CaMKII*α*‐hCHR2 (E123T/T159C)‐mCherry‐WPRE‐hGH pA (4.91 × 10^12^ genomic copies mL^−1^, BrainVTA, China) or rAAV‐CaMKII*α*‐mCherry‐WPRE‐hGH pA (2.70 × 10^12^ genomic copies mL^−1^, BrainVTA, China) was bilaterally injected into the mPFC. For chemogenetic stimulation, 200 nL of rAAV‐CaMKII*α*‐hM3D‐(Gq)‐mCherry‐WPRE‐hGH pA (5.29 × 10^12^ genomic copies mL^−1^, BrainVTA, China) or rAAV‐CaMKII*α*‐mCherry‐WPRE‐hGH pA (5.88 × 10^12^ genomic copies/ml, BrainVTA, China) was bilaterally injected into the same location. For *Wnt2* overexpression, 200 nL of rAAV‐CaMKII*α*‐Wnt2‐EGFP‐3Flag‐SV40 pA (4.13 × 10^13^ genomic copies mL^−1^, GeneChem, China) or rAAV‐CaMKII*α*‐EGFP‐3Flag‐SV40 pA (2.7 × 10^13^ genomic copies mL^−1^, GeneChem, China) was bilaterally injected as previously described.

### Optogenetic and Chemogenetic Stimulation

For optogenetic stimulation, an optical fiber (OD of 200 µm, NA of 0.50, Inper, China) was implanted 10 µm above the center of the two viral injection sites 3 weeks after virus injection. Dental cement was used to secure the optic fiber to the skull. The mice were anaesthetized with 3% isoflurane in 33% oxygen before fiber implantation and treated with carprofen (5.0 mL kg^−1^) postoperatively to reduce pain. Ten days later, the implanted optic fiber was connected to a pulse‐modulated generator (Inper, China) with fiber sleeves to transmit blue light (473 nm, 5 mW, 20 Hz). Stimulation was administered for 30 min per day for 14 consecutive days. For chemogenetic stimulation, 1 month after virus injection, CNO (1 mg kg^−1^, Tocris, UK) was intraperitoneally injected daily for 14 consecutive days.

### Behavioral Experiments

Experiments were performed during the light cycle (7 AM to 7 PM). The mice were handled by the investigator for 5 min per day from three days before the experiments until the end of the experiments. The procedure was performed in the holding room in which the mice were housed. After each experiment, the mice were returned to their home cages. All behavioral data were analyzed by investigators blinded to the experimental groups. The open field test (OFT) was used to measure locomotor activity and anxiety‐like behavior. During the OFT, each mouse was gently placed in the center of an open chamber (40 × 40 × 50 cm). A camera was installed above the test chamber to record the movement trajectories of the mice for 10 min. The total distance travelled and time spent in the center zone and corner zone were analyzed with ANY‐maze software (Stoelting, USA). The chamber was cleaned using 75% ethanol after each trial. The Y‐maze test and T‐maze test were both performed to assess short‐term working memory as previously described.^[^ ^43^ ^]^ The Y‐maze consisted of three arms of equal size (40 cm long, 10 cm wide, and 12 cm high), with an angle of 120° between adjacent arms. The mice were placed in the center of the three arms and allowed to move freely through the maze for 8 min. The sequence of arm entries was recorded, and the percentage of spontaneous alternations was calculated as the proportion of times that all three arms were entered consecutively. Before each trial, the maze was cleaned with 75% ethanol. The T‐maze consisted of two goal arms (30 cm long, 10 cm wide, and 10 cm high) and one vertical start arm (30.7 cm long, 13 cm wide, and 10 cm high). The test procedure included the habituation, training, and trial phases. During the two‐day habituation phase, mice received approximately 20 reward pellets in their home cages after 5 min of handling per day for taste habituation and hyponeophagia elimination. After that, the mice were subjected to two days of training. First, the mice were fasted for at least 6 h. Then, a certain number of reward pellets were placed in the food wells in the two goal arms, and a mouse was placed in the start arm and allowed to enter one of the goal arms (the other goal arm was blocked by a door) and consume all the food. After the mouse consumed the food, it was removed from the maze for 15 s. During this time, the door was removed, and the maze was cleaned with 75% ethanol. The mouse was then placed in the start arm and allowed to choose a goal arm. If the mouse chose the correct goal arm (the one that had been blocked previously), it received a food reward. Otherwise, the mouse was removed after a period of time equivalent to that required to consume all food. Each mouse underwent 4 training trials, with 15 min between consecutive trainings. Finally, the trial phase, which was identical to the training phase, was performed. Each mouse underwent 10 trials, with 15 min between each trial. The percentage of spontaneous alternations was calculated as the proportion of times that the mouse entered the correct goal arm.

### Electrophysiological Recordings

Slices (300 µm) of the mPFC and corpus callosum were cut using a Vibroslice instrument (Leica VT 1000s) in oxygenated (95% O_2_/5% CO_2_) iced cutting solution containing (in mm) 120 choline Cl‐, 2.5 KCl, 7 MgCl_2_, 0.5 CaCl_2_, 1.25 NaH_2_PO_4_, 25 NaHCO_3_, 10 glucose, 5 Na^+^ ascorbate, and 3Na^+^ pyruvate. Then, the slices were incubated in cutting solution for 15 min at 34 °C and transferred to oxygenated artificial CSF (ACSF) containing (in mm) 124 NaCl, 2.5 KCl, 2 MgSO_4_, 2.5 CaCl_2_, 1.25 NaH2PO_4_, 25 NaHCO_3_, and 10 glucose at room temperature (25 °C) for 1 h before recording. Whole‐cell patch clamp recordings (MultiClamp 700B amplifier, Digidata 1550B analogue‐to‐digital converter) and pClamp 10.7 software (Molecular Devices, USA) were used to record neuronal action potentials as previously described.^[^ ^44^ ^]^ Briefly, glass pipettes (3–5 MΩ) filled with internal solution (125 mm potassium gluconate, 10 mm HEPES buffer, 5 mm KCl, 1 mm MgCl_2_·6H_2_O, 10 Na_2_‐phosphocreatine, 4 mm Mg‐ATP, 0.3 mm Na‐GTP, and 0.2 mm EGTA; pH adjusted to 7.2 with KOH; 285 mOsm) were used. Evoked action potentials were recorded in current clamp mode. Pyramidal neurons in the mPFC were stimulated by a series of 500 ms depolarizing current pulses (from 0 to 140 pA, step of 20 pA). The minimum depolarizing current needed to induce an action potential was used as the threshold current for spike generation. For miniature excitatory postsynaptic current (mEPSC) recording, pyramidal neurons in the mPFC were held at −70 mV in the presence of the GABAAR antagonist bicuculline (20 µm) and the Na^+^ channel blocker TTX (1 µm). For CAP recordings, acute slices containing the corpus callosum were transferred to a microelectrode array, continuously perfused with oxygenated ACSF (2 mL min^−1^) and maintained at 32 °C for recording. CAPs in the corpus callosum were recorded using the MEA‐2100‐60‐System (Multi Channel Systems, Germany). CAPs were recorded using LTP‐Director software (Multi Channel Systems, Germany). Input–output curves were generated by varying the intensity of the stimulation from 10 to 130 µA (step of 30 µA). LTP‐Analyser (Multi Channel Systems, Germany) software was used for data analysis. The myelinated fiber‐response amplitude was defined as the voltage difference from the first peak to the first trough (N1).

### MRI Data Acquisition and Analysis

MRI data were obtained on a 9.4T Bruker MR system (BioSpec 94/20 USR, Bruker) using a 440‐mT m^−1^ gradient set an 86‐mm volume transit RF coil and a single channel surface head coil. The mice were anaesthetized by inhalation of 3% isoflurane before scanning, and physiological parameters were monitored and kept constant during the experiment. Tooth and ear bars were used to restrain the mice for imaging. A 2D rapid acquisition with relaxation enhancement (RARE) sequence was applied to obtain T2‐weighted images with the following parameters: repetition time (TR): 2500 ms; echo time (TE): 33 ms; field of view (FOV): 20 mm × 20 mm; matrix: 256 × 256; 22 adjacent slices of 0.7 mm slice thickness. The spin‐echo echo‐planar imaging (SE‐EPI) sequence was used to obtain diffusion‐weighted images with the following parameters: two *b*‐values (*b* = 0 and 1000 s mm^−2^) along with 30 noncollinear directions; *δ* = 4.1 ms; *Δ* = 10.3 ms; TR: 1500 ms; TE: 23.27 ms; FOV: 20 mm × 20 mm; matrix: 128 × 128; 22 adjacent slices of 0.7 mm slice thickness. Then, MRIcron was used to transform the imaging data into NIFTI format. Diffusion data were postprocessed using the FSL (v.5.0.9) pipeline, including corrections for eddy currents and movement artefacts (*eddy_correct*), rotations of gradient directions according to eddy current corrections (*fdt_rotate_bvecs*), brain mask extractions based on b0 images (*bet*), and FA map calculations by fitting a diffusion tensor model at each voxel (*dtifit*). Chemogenetic‐related data were normalized to the mean value of the sham group, and *Wnt2‐*related data were normalized to the mean value of the control group. The data were analyzed, and heatmaps were generated by the Institute of High Energy Physics, Chinese Academy of Sciences.

### Black‐Gold Staining

The mice were transcardially perfused with 4% paraformaldehyde and 0.9% saline. The brain was dehydrated in 15% and 30% sucrose for 24 h each and cut into 20 µm coronal sections with a cryostat microtome (Leica, Wetzlar, Germany). Black‐gold staining was performed using a Black‐Gold II Myelin Staining Kit (Biosensis, USA) following the manufacturer's instructions. Briefly, 0.3% Black‐gold II and 1% sodium thiosulfate solutions were preheated to 65 °C. Afterward, the brain slices were rehydrated and incubated with Black‐gold II solution for approximately 12 min at 65 °C until the finest myelinated fibers turned dark red. Then, the slices were washed with distilled water and incubated with sodium thiosulfate solution for 3 min at 65 °C. Subsequently, slices were washed with distilled water again, dehydrated in a series of graded ethanol solutions and cleared in xylene for 2 min. Finally, the slices were sealed with neutral balsam. Images were captured using a light microscope (Olympus BX51, Japan) and analyzed with ImageJ software.

### Immunofluorescence

Rehydrated brain slices were permeabilized in 0.1% Triton X‐100 for 20 min, blocked with 2% BSA for 1 h at room temperature, and immunostained with primary antibodies against MAG (Abcam, USA, ab89780), MBP (Abcam, USA, ab7349), APC‐CC1 (Millipore, USA, ABN899), Olig2 (Calbiochem, USA, OP80), Wnt2 (Abcam, USA, ab109222), Ki67 (Abclonal, China, A2094), *β*‐catenin (Proteintech, China, 51067‐2‐AP), CamkII*α* (Invitrogen, USA, MA1‐048), Iba1 (Abcam, USA, ab178846), GFAP (Bioworld, USA, BS6460) and NF‐H (Proteintech, China, 60331) at 4 °C overnight. On the second day, the slices were washed three times with phosphate‐buffered saline (PBS) and incubated with secondary antibodies (Invitrogen, USA) for 1.5 h at room temperature in the dark. The cell nuclei were then stained with DAPI (1 µg mL^−1^) for 15 min. Finally, images were obtained with a confocal fluorescence microscope (Olympus FV3000, Japan). All images were analyzed with ImageJ software.

### EdU Administration and Staining

EdU (20 mg kg^−1^, Invitrogen, CA) was intraperitoneally injected daily for the first two days or 14 continuous days during optogenetic/chemogenetic manipulation, and the brain samples were collected after neuronal stimulation. EdU staining of brain tissue was conducted using a Click‐iT EdU Imaging Kit (Invitrogen, CA) according to the manufacturer's instructions. Briefly, slices were fixed with 4% paraformaldehyde in PBS for 15 min after being thawed at room temperature. Next, the sections were washed twice with 3% bovine serum albumin (BSA) in PBS and permeabilized with 0.5% Triton X‐100 in PBS for 20 min. The slices were again washed twice with 3% BSA in PBS and then incubated with Click‐iT reaction cocktail containing Click‐iT reaction buffer, CuSO_4_, Alexa Fluor 647‐conjugated azide, and reaction buffer additive for 30 min. The sections were washed once more with PBS. All steps were carried out at room temperature.

### TUNEL Staining

TUNEL staining was performed using TUNEL BrightGreen Apoptosis Detection Kit (Vazyme, China, A112) according to manufacturer's instructions. Briefly, permeabilized brain slices were blocked with 2% BSA for 1 h, incubated with 1× Equilibration Buffer for 20 min at room temperature, and incubated with TdT working solution for 1 h at 37 °C. After washing, the slices were incubated with the primary antibody against Olig2 (Calbiochem, USA, OP80) for downstream immunofluorescence assay.

### Western Blotting

Western blotting was performed as previously described.^[^ ^45^ ^]^ Briefly, proteins were homogenized using RIPA lysis buffer (Bioworld, USA), and the protein concentration was quantified by a BCA protein assay kit (Invitrogen, USA). Then, equal amounts of protein were separated by 10% or 12% (sodium dodecyl sulfate–polyacrylamide gel electrophoresis) SDS–PAGE and transferred to polyvinylidene fluoride membranes. The membranes were blocked in 5% milk for 2 h and incubated at 4 °C overnight with the following primary antibodies: anti‐MAG (Cell Signaling, USA, ab89780), anti‐MBP (Abcam, USA, ab7349), anti‐*β*‐catenin (Proteintech, CHINA, 51067‐2‐AP), and anti‐*β*‐actin (Bioworld, USA, AP0060). Subsequently, the membranes were incubated in secondary antibodies for 2 h at room temperature and then visualized using a Gel‐Pro System (Tanon Technologies, China). The density of the protein bands was quantified using ImageJ software (ImageJ 1.5, NIH, USA).

### Transmission Electron Microscopy

Randomly selected mice from each group were anaesthetized with isoflurane and perfused with PBS. The corpus callosum was quickly dissociated and soaked overnight in 2.5% glutaraldehyde (EM grade). Sample preparation and image acquisition were performed by Servicebio (China). The extent of axonal myelination was quantified by calculating the g‐ratio (the ratio of the axonal diameter to the myelin sheath thickness) as described above. ImageJ software was used for measurement of the axonal caliber and axonal counting.

### Real‐Time PCR (RT–PCR)

Total RNA was isolated using TRIzol (Invitrogen, USA) as previously described.^[^ ^46^ ^]^ DNA wiper mix was added to remove genomic DNA, and a PrimeScript RT Reagent Kit (Vazyme, Nanjing, China) was used according to the manufacturer's instructions. Complementary DNA synthesis was also performed using the PrimeScript RT Reagent Kit according to the manufacturer's instructions. RT–PCR was conducted on the Step One Plus PCR system (Applied Biosystems, Foster City, CA, USA) in a 20 µL reaction mixture using a SYBR Green Kit (Applied Biosystems). The primer sequences used for the experiment were as follows:

*Lamb1*‐F: AGACTTTGGGGGTTCATGTCA

*Lamb1*‐R: ATCGTCCCGTCTCCTTGTCA

*Wnt2*‐F: CTCGGTGGAATCTGGCTCTG

*Wnt2*‐R: CACATTGTCACACATCACCCT

*Adamts3*‐F: GCTGGTACTGACGAAATGGTG

*Adamts3*‐R: ATGACACGTCCCTGGGTGA

*Nppa*‐F: GCTTCCAGGCCATATTGGAG

*Nppa*‐R: GGGGGCATGACCTCATCTT

*Mdga1*‐F: ATGGAGGTGACCTGTCTTCTAC

*Mdga1*‐R: GGCTGGAGCATAGACTCCTT

*Lrrc55*‐F: AGCGTAAAAGAAGGGGCACA

*Lrrc55*‐R: AGCTTCCGAACAGATTGCCT

*Sez6*‐F: TCTGTGCAATAACAACATCTCGG

*Sez6*‐R: GCTGTAGGTGCAATCTAGGAGC

*Mbl2*‐F: TGACAGTGGTTTATGCAGAGAC

*Mbl2*‐R: CGTCACGTCCATCTTTGCC

*Ptgis*‐F: ACAGCATCAAACAATTTGTCGTC

*Ptgis*‐R: GCATCAGACCGAAGCCATATCT

*Cdh13*‐F: CTGTGGGGGTCATTGTCAACT

*Cdh13*‐R: GTTGGTCTGTGGGTTGGTGT

*Tac1*‐F: CAGTCACCAACTCAGTCCTGC

*Tac1*‐R: CACAACGATCTCGAAGTCCCC

*Ntm*‐F: AAAACCATCCAGGCAAAAATGC

*Ntm*‐R: TAGGCTCCGGTCTACCTGTG

*Thbs1*‐F: GGGGAGATAACGGTGTGTTTG

*Thbs1*‐R: CGGGGATCAGGTTGGCATT

*Lrfn55*‐F: TGTTTCTCATTGGCATAGCTGT

*Lrfn55*‐R: TGGTGGAACAAATAGAAGCCCT

*Nrg3*‐F: TTACGCTGTAGCGACTGCATC

*Nrg3*‐R: GCCTACCACGATCCATTTAAGC

*Scgb3a1*‐F: GCTTTCTTCATGGACTCATTGGC

*Scgb3a*1‐R: GGGCTTAATGGTAGGCTAGGCA

*Nrn1*‐F: GGCTTTGCTGAATGTCTCATCC

*Nrn1*‐R: CAATCCGCTCCCGAACACT

*Gapdh*‐F: AGGTCGGTGTGAACGGATTTG

*Gapdh*‐R: TGTAGACCATGTAGTTGAGGTCA

### Microarray Analysis

Total RNA was extracted from mPFC tissues using TRIzol, and the RNA was reverse transcribed into cDNA (Takara, Dalian, China). Then, cDNA was transcribed into cRNA labelled with Cyanine‐3‐CTP (Cy3) (Agilent, USA). The microarray was scanned using an Agilent Microarray Scanner (Agilent, USA) after fragmentation, hybridization, and washing. RNAs with differential expression were identified using a whole genome microarray (fold change > 2, *p* < 0.05). Microarray analysis was performed using Agilent Feature Extraction by Oebiotech (China).

### Cell Culture

Primary cortical neurons were prepared from embryonic day (E)15–17 B6 mouse embryos as previously described,^[^ ^47^ ^]^ cultured in neurobasal medium supplemented with B27 (Invitrogen, USA) and 25 nm glutamine at 37 °C in a humidified atmosphere containing 5% CO_2_ and then collected for subsequent experiments. Half of the cell medium was exchanged every two days. Primary glial cells were prepared from 1‐day‐old C57/BL6J mice as previously described.^[^ ^30^ ^]^ Primary microglia and astrocytes were cultured in Dulbecco's modified Eagle's medium (Invitrogen, USA) supplemented with 10% fetal bovine serum (HyClone, Logan, UT, USA) and 100 U mL^−1^ antibiotics at 37 °C in a humidified 5% CO_2_ incubator. After 10 days of culture, the flasks were shaken at 180 rpm for 10 min to separate the primary microglia and astrocytes. Then, the cells were collected for further study. Primary OPCs were cultured in DMEM F12 (Invitrogen, USA) supplemented with 2% B27, 100 U mL^−1^ antibiotics, 30 ng mL^−1^ PDGF (Thermo Fisher Scientific, USA), and 10 ng mL^−1^ FGF (Thermo Fisher Scientific, USA) for 6 days. Afterward, 50 ng mL^−1^ T3 (Thermo Fisher Scientific, USA), 200 ng mL^−1^ recombinant Wnt2 protein (Cusabio, China), 100 ng mL^−1^ recombinant Dkk1 protein (Cusabio, China) and basic medium DMEM F12 supplemented with 2% B27, 100 U mL^−1^ antibiotics, and 20 ng mL^−1^ CNTF (Thermo Fisher Scientific, USA) were applied to induce differentiation. OPCs were divided into the following five groups and cultured with the indicated medium: the control group (basic medium), T3 group (basic medium containing T3), T3+Wnt2 group (basic medium containing T3 and wnt2), T3+Dkk1 group (basic medium containing T3 and Dkk1), and T3+Wnt2+Dkk1 group (basic medium containing T3, Wnt2 and Dkk1). After 6 days, all the samples were collected for further study. Half of the cell medium was exchanged every two days.

### Data Analysis and Statistics

SPSS software version 22.0 (SPSS Inc., USA) and Prism 8 software (GraphPad Software, USA) were used to perform the statistical analysis. Numerical data are expressed as the mean ± SEM. Student's *t*‐test or the Mann–Whitney test was used to evaluate statistical significance between two groups. The statistical significance of data with one factor among multiple groups was analyzed by one‐way analysis of variance (ANOVA) followed by Tukey's post‐hoc test or the Kruskal–Wallis test followed by Tukey's post‐hoc test, and the statistical significance of data with two factors was analyzed by two‐way ANOVA followed by Tukey's post‐hoc test. Correlation analyses were performed using Pearson's correlation. Detailed statistical methods are provided in each figure legend. Differences were considered statistically significant at *p* < 0.05.

## Conflict of Interest

The authors declare no conflict of interest.

## Author Contributions

S.D., S.S., and L.Z. contributed equally to this work. P.L. and Y.X. conceived the study and designed the experiments; S.D., S.S., L.Z., and H.L. performed the experiments; S.D., S.S., L.Z., S.X., H.L., X.C., and X.B. analyzed and contributed reagents/materials/analysis tools; S.D., S.S., P.L. wrote the paper and Y.X. revised the paper. All authors reviewed the final manuscript.

## Supporting information

Supporting Information

## Data Availability

The data that support the findings of this study are available from the corresponding author upon reasonable request.

## References

[1] J. M. Wardlaw , C. Smith , M. Dichgans , Lancet Neurol. 2019, 18, 684. 31097385 10.1016/S1474-4422(19)30079-1

[2] a) H. Söderlund , L. Nyberg , R. Adolfsson , L. G. Nilsson , L. J. Launer , Cortex 2003, 39, 1093; 14584568 10.1016/s0010-9452(08)70879-7

[3] a) J. M. Wardlaw , E. E. Smith , G. J. Biessels , C. Cordonnier , F. Fazekas , R. Frayne , R. I. Lindley , J. T. O'Brien , F. Barkhof , O. R. Benavente , S. E. Black , C. Brayne , M. Breteler , H. Chabriat , C. Decarli , F. E. de Leeuw , F. Doubal , M. Duering , N. C. Fox , S. Greenberg , V. Hachinski , I. Kilimann , V. Mok , R. Oostenbrugge , L. Pantoni , O. Speck , B. C. Stephan , S. Teipel , A. Viswanathan , D. Werring , et al., Lancet Neurol. 2013, 12, 822; 23867200 10.1016/S1474-4422(13)70124-8 PMC3714437

[4] R. P. Kloppenborg , P. J. Nederkoorn , M. I. Geerlings , E. van den Berg , Neurology 2014, 82, 2127. 24814849 10.1212/WNL.0000000000000505

[5] H. Y. Hu , Y. N. Ou , X. N. Shen , Y. Qu , Y. H. Ma , Z. T. Wang , Q. Dong , L. Tan , J. T. Yu , Neurosci. Biobehav. Rev. 2021, 120, 16. 33188821 10.1016/j.neubiorev.2020.11.007

[6] N. D. Prins , P. Scheltens , Nat. Rev. Neurol. 2015, 11, 157. 25686760 10.1038/nrneurol.2015.10

[7] a) B. F. Verhaaren , M. W. Vernooij , R. de Boer , A. Hofman , W. J. Niessen , A. van der Lugt , M. A. Ikram , Hypertension 2013, 61, 1354; 23529163 10.1161/HYPERTENSIONAHA.111.00430

[8] a) J. D. Williamson , L. J. Launer , R. N. Bryan , L. H. Coker , R. M. Lazar , H. C. Gerstein , A. M. Murray , M. D. Sullivan , K. R. Horowitz , J. Ding , S. Marcovina , L. Lovato , J. Lovato , K. L. Margolis , C. Davatzikos , J. Barzilay , H. N. Ginsberg , P. E. Linz , M. E. Miller , JAMA Intern. Med. 2014, 174, 324;24493100

[9] a) M. Simons , K. A. Nave , Cold Spring Harbor Perspect. Biol. 2015, 8, a020479; 10.1101/cshperspect.a020479 PMC4691794 26101081

[10] a) K. A. Nave , H. B. Werner , Annu. Rev. Cell Dev. Biol. 2014, 30, 503; 25288117 10.1146/annurev-cellbio-100913-013101

[11] S. E. Pease‐Raissi , J. R. Chan , Neuron 2021, 109, 1258. 33621477 10.1016/j.neuron.2021.02.003 PMC8068592

[12] E. M. Gibson , D. Purger , C. W. Mount , A. K. Goldstein , G. L. Lin , L. S. Wood , I. Inema , S. E. Miller , G. Bieri , J. B. Zuchero , B. A. Barres , P. J. Woo , H. Vogel , M. Monje , Science 2014, 344, 1252304. 24727982 10.1126/science.1252304 PMC4096908

[13] T. Ben‐Shaanan , M. Schiller , A. Rolls , Brain Behav. Immunol. 2017, 65, 27890661. 10.1016/j.bbi.2016.11.024 27890661

[14] K. Deisseroth , Nat. Methods 2011, 8, 26. 21191368 10.1038/nmeth.f.324 PMC6814250

[15] a) A. A. Gershon , P. N. Dannon , L. Grunhaus , Am. J. Psychiatry 2003, 160, 835; 12727683 10.1176/appi.ajp.160.5.835

[16] M. Shibata , R. Ohtani , M. Ihara , H. Tomimoto , Stroke 2004, 35, 2598. 15472111 10.1161/01.STR.0000143725.19053.60

[17] H. Takao , N. Hayashi , K. Ohtomo , Neuroscience 2013, 231, 23219841. 10.1016/j.neuroscience.2012.11.038 23219841

[18] M. M. Reimer , J. McQueen , L. Searcy , G. Scullion , B. Zonta , A. Desmazieres , P. R. Holland , J. Smith , C. Gliddon , E. R. Wood , P. Herzyk , P. J. Brophy , J. McCulloch , K. Horsburgh , J. Neurosci. 2011, 31, 18185. 22159130 10.1523/JNEUROSCI.4936-11.2011 PMC4337974

[19] a) C. D. Langen , L. G. M. Cremers , M. de Groot , T. White , M. A. Ikram , W. J. Niessen , M. W. Vernooij , NeuroImage 2018, 183, 745; 30144572 10.1016/j.neuroimage.2018.08.037

[20] R. W. Mangum , J. S. Miller , W. S. Brown , A. A. T. Nolty , L. K. Paul , J. Int. Neuropsychol. Soc. 2021, 27, 1037. 33749569 10.1017/S1355617721000096 PMC8744483

[21] a) Z. Wang , L. Bai , Q. Liu , S. Wang , C. Sun , M. Zhang , Y. Zhang , Ann. Clin. Transl. Neurol. 2020, 7, 2409; 33119959 10.1002/acn3.51231 PMC7732249

[22] P. Shrestha , A. Mousa , N. Heintz , Elife 2015, 4, 26371510. 10.7554/eLife.08752 PMC4566133 26371510

[23] Y. D. Reijmer , A. P. Schultz , A. Leemans , M. J. O'Sullivan , M. E. Gurol , R. Sperling , S. M. Greenberg , A. Viswanathan , T. Hedden , NeuroImage 2015, 117, 222. 26025290 10.1016/j.neuroimage.2015.05.054 PMC4511724

[24] K. Zilles , Anatomy of the Neocortex: Cytoarchitecture and Myeloarchitecture, Cambridge, MA, MIT 1990 pp. 77–113.

[25] a) H. G. Bae , T. K. Kim , H. Y. Suk , S. Jung , D. G. Jo , Arch. Pharm. Res. 2020, 43, 920; 32975736 10.1007/s12272-020-01270-x

[26] a) M. Naruse , Y. Ishizaki , K. Ikenaka , A. Tanaka , S. Hitoshi , J. Physiol. Sci. 2017, 67, 63; 27573166 10.1007/s12576-016-0479-7 PMC5368213

[27] a) P. E. Steadman , F. Xia , M. Ahmed , A. J. Mocle , A. R. A. Penning , A. C. Geraghty , H. W. Steenland , M. Monje , S. A. Josselyn , P. W. Frankland , Neuron 2020, 105, 150; 31753579 10.1016/j.neuron.2019.10.013 PMC7579726

[28] A. C. Geraghty , E. M. Gibson , R. A. Ghanem , J. J. Greene , A. Ocampo , A. K. Goldstein , L. Ni , T. Yang , R. M. Marton , S. P. Paşca , M. E. Greenberg , F. M. Longo , M. Monje , Neuron 2019, 103, 250. 31122677 10.1016/j.neuron.2019.04.032 PMC6697075

[29] F. C. Ortiz , C. Habermacher , M. Graciarena , P. Y. Houry , A. Nishiyama , B. Nait Oumesmar , M. C. Angulo , JCI Insight 2019, 5, 30896448. 10.1172/jci.insight.123434 PMC6538342 30896448

[30] N. Miyamoto , S. Magami , T. Inaba , Y. Ueno , K. Hira , C. Kijima , S. Nakajima , K. Yamashiro , T. Urabe , N. Hattori , Glia 2020, 68, 1910. 32108971 10.1002/glia.23814

[31] Y. Xie , X. Zhang , P. Xu , N. Zhao , Y. Zhao , Y. Li , Y. Hong , M. Peng , K. Yuan , T. Wan , R. Sun , D. Chen , L. Xu , J. Chen , H. Guo , W. Shan , J. Li , R. Li , Y. Xiong , D. Liu , Y. Wang , G. Liu , R. Ye , X. Liu , J. Clin. Invest. 2021, 131, 33141760. 10.1172/JCI128114 PMC7773390 33141760

[32] a) C. L. Cullen , M. Senesi , A. D. Tang , M. T. Clutterbuck , L. Auderset , M. E. O'Rourke , J. Rodger , K. M. Young , Glia 2019, 67, 1462; 30989733 10.1002/glia.23620 PMC6790715

[33] a) Z. Shao , X. Lee , G. Huang , G. Sheng , C. E. Henderson , D. Louvard , J. Sohn , B. Pepinsky , S. Mi , J. Neurosci. 2017, 37, 3127; 28193690 10.1523/JNEUROSCI.3722-16.2017 PMC6596780

[34] L. A. Osso , K. A. Rankin , J. R. Chan , Neuron 2021, 109, 3619. 34536353 10.1016/j.neuron.2021.08.015 PMC8602781

[35] E. Stanganello , E. E. Zahavi , M. Burute , J. Smits , I. Jordens , M. M. Maurice , L. C. Kapitein , C. C. Hoogenraad , iScience 2019, 13, 318. 30878878 10.1016/j.isci.2019.02.029 PMC6423405

[36] G. A. Wayman , S. Impey , D. Marks , T. Saneyoshi , W. F. Grant , V. Derkach , T. R. Soderling , Neuron 2006, 50, 897. 16772171 10.1016/j.neuron.2006.05.008

[37] A. J. Langseth , R. N. Munji , Y. Choe , T. Huynh , C. D. Pozniak , S. J. Pleasure , J. Neurosci. 2010, 30, 13367. 20926663 10.1523/JNEUROSCI.1934-10.2010 PMC2954511

[38] a) S. P. Fancy , S. E. Baranzini , C. Zhao , D. I. Yuk , K. A. Irvine , S. Kaing , N. Sanai , R. J. Franklin , D. H. Rowitch , Genes Dev. 2009, 23, 1571; 19515974 10.1101/gad.1806309 PMC2704469

[39] a) S. P. Fancy , E. P. Harrington , T. J. Yuen , J. C. Silbereis , C. Zhao , S. E. Baranzini , C. C. Bruce , J. J. Otero , E. J. Huang , R. Nusse , R. J. Franklin , D. H. Rowitch , Nat. Neurosci. 2011, 14, 1009; 21706018 10.1038/nn.2855 PMC3145042

[40] M. Tawk , J. Makoukji , M. Belle , C. Fonte , A. Trousson , T. Hawkins , H. Li , S. Ghandour , M. Schumacher , C. Massaad , J. Neurosci. 2011, 31, 3729. 21389228 10.1523/JNEUROSCI.4270-10.2011 PMC6622795

[41] Z. M. Dai , S. Sun , C. Wang , H. Huang , X. Hu , Z. Zhang , Q. R. Lu , M. Qiu , J. Neurosci. 2014, 34, 8467. 24948802 10.1523/JNEUROSCI.0311-14.2014 PMC4147624

[42] Y. Liu , Y. Wang , W. Yuan , F. Dong , F. Zhen , J. Liu , L. Yang , X. Qu , R. Yao , Neurol. Res. 2021, 43, 543. 33616025 10.1080/01616412.2021.1888604

[43] a) R. M. Deacon , J. N. Rawlins , Nat. Protoc. 2006, 1, 7; 17406205 10.1038/nprot.2006.2

[44] M. Chen , S. Shu , H. H. Yan , L. Pei , Z. F. Wang , Q. Wan , L. L. Bi , Neuropharmacology 2017, 118, 242. 28302570 10.1016/j.neuropharm.2017.03.014

[45] P. Y. Liu , Z. Zhang , Y. Liu , X. L. Tang , S. Shu , X. Y. Bao , Y. Zhang , Y. Gu , Y. Xu , X. Cao , Front. Cell Neurosci. 2019, 13, 360. 31447648 10.3389/fncel.2019.00360 PMC6691060

[46] J. Pan , J. L. Jin , H. M. Ge , K. L. Yin , X. Chen , L. J. Han , Y. Chen , L. Qian , X. X. Li , Y. Xu , J. Neuroinflammation 2015, 12, 51. 25889216 10.1186/s12974-015-0270-3 PMC4378556

[47] W. Tao , L. Yu , S. Shu , Y. Liu , Z. Zhuang , S. Xu , X. Bao , Y. Gu , F. Cai , W. Song , Y. Xu , X. Zhu , Mol. Ther. 2021, 29, 396. 32950103 10.1016/j.ymthe.2020.09.006 PMC7791017

