# Supplementary material for: Optogenetic Stimulation of mPFC Alleviates White Matter Injury‐Related Cognitive Decline after Chronic Ischemia through Adaptive Myelination
Source: Adv Sci (Weinh). 2022 Dec 18;10(5):2202976. doi: 10.1002/advs.202202976 (PMC9929132; doi:10.1002/advs.202202976)
Supplement: Supplementary file 1 — Supporting Information [file ADVS-10-2202976-s001.pdf]

ADVANCED SCIENCE
Open Access

Supporting Information

for *Adv. Sci.*, DOI 10.1002/advs.202202976

Optogenetic Stimulation of mPFC Alleviates White Matter Injury-Related Cognitive Decline after Chronic Ischemia through Adaptive Myelination

Shiji Deng, Shu Shu, Lili Zhai, Shengnan Xia, Xiang Cao, Huiya Li, Xinyu Bao, Pinyi Liu and Yun Xu**

Supporting Information

Optogenetic stimulation of mPFC alleviates white matter injury-related cognitive decline after chronic ischemia through adaptive myelination

Shiji Deng, Shu Shu, Lili Zhai, Shengnan Xia, Xiang Cao, Huiya Li, Xinyu Bao, Pinyi Liu, and Yun Xu**

Figure. S1

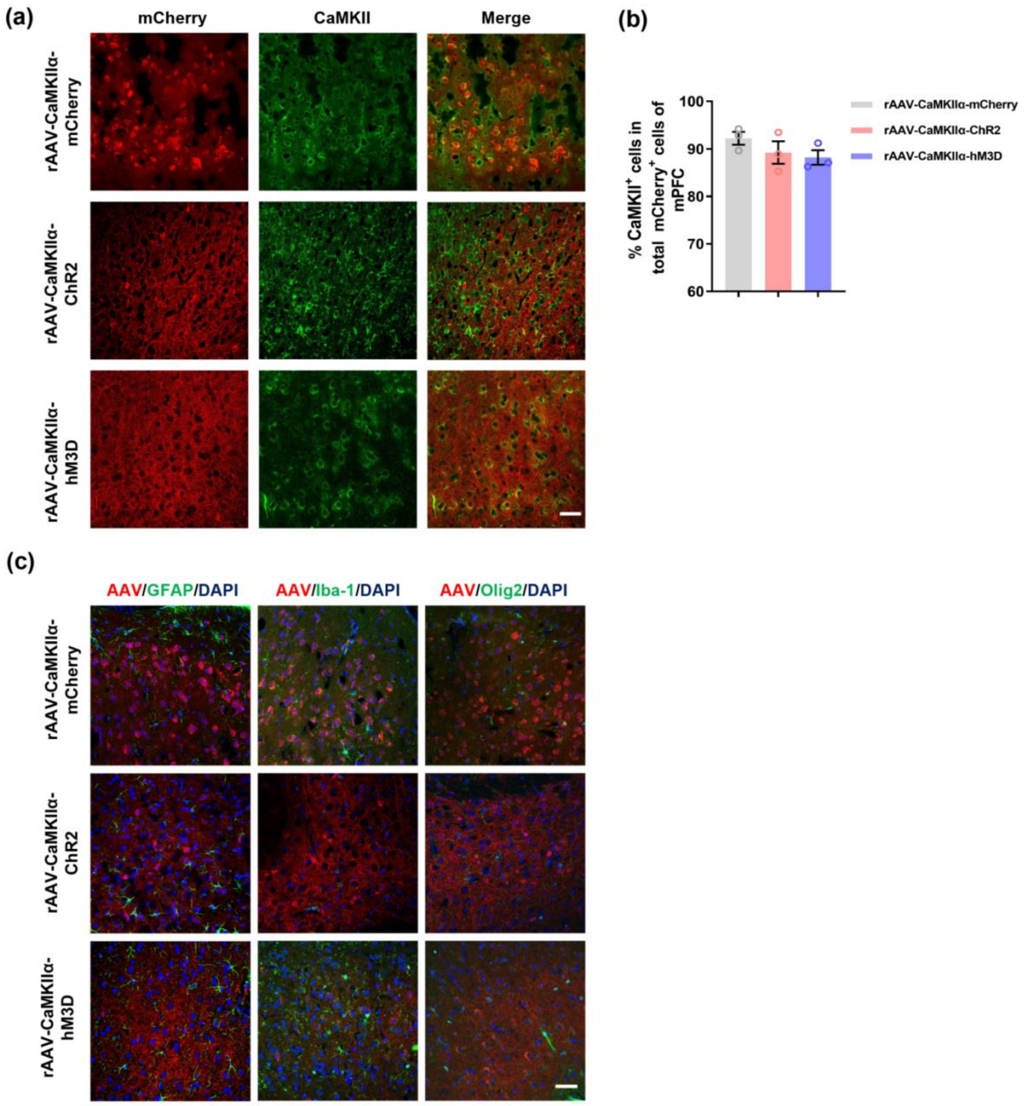

Figure S1. The transduction efficiency and specificity of adeno-associated virus. a) Representative immunostaining of CaMKII and rAAV-associated mCherry expression in the mPFC in the BCAS mCherry, BCAS ChR2 groups, and BCAS hM3D groups at 2 months after surgery. Scale bar: 50 µm. b) Proportion of CaMKII$^+$/mCherry$^+$ cells in the mPFC in the BCAS mCherry, BCAS ChR2 groups, and BCAS hM3D groups at 2 months after surgery. *n*=3 per group. c) Representative immunostaining of GFAP, Iba1, Olig2, and rAAV-associated mCherry expression in the mPFC in the BCAS mCherry, BCAS ChR2 groups, and BCAS hM3D groups at 2 months after surgery. Scale bar: 50 µm. The data are presented as the mean ± SEM.

Figure. S2

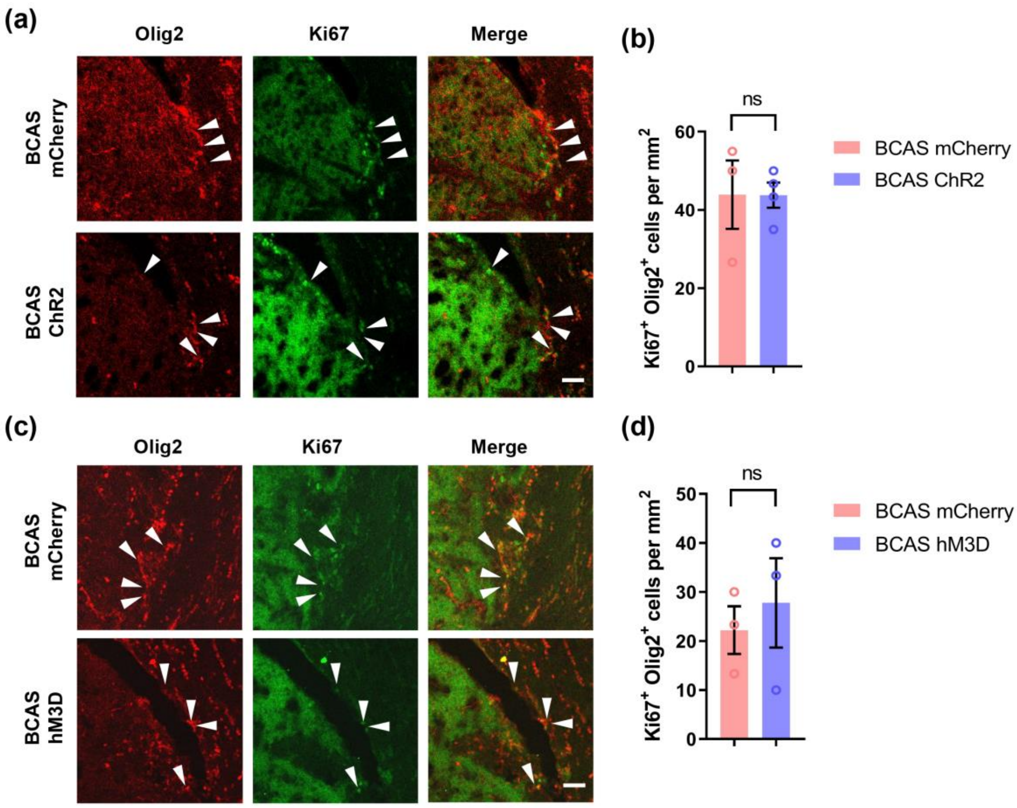

Figure S2. Neuromodulation of mPFC does not affect the proliferation of OPC. a) Representative immunostaining of Olig2 and Ki67 in the subventricular zone (SVZ) in the BCAS mCherry and BCAS ChR2 groups at 2 months after surgery. Scale bar: 25 µm. b) Density of $Ki67^+$ $Olig2^+$ cells in the SVZ in the BCAS mCherry and BCAS ChR2 groups at 2 months after surgery. *n*=3-4 per group. c) Representative immunostaining of Olig2 and Ki67 in the SVZ in the BCAS mCherry and BCAS hM3D groups at 2 months after surgery. Scale bar: 25 µm. d) Density of $Ki67^+$ $Olig2^+$ cells in the subventricular zone in the BCAS mCherry and BCAS hM3D groups at 2 months after surgery. *n*=3 per group. The data are presented as the mean ± SEM. *P* values were determined by Student's t test in (b) and (d). ns, $P > 0.05$.

Figure. S3

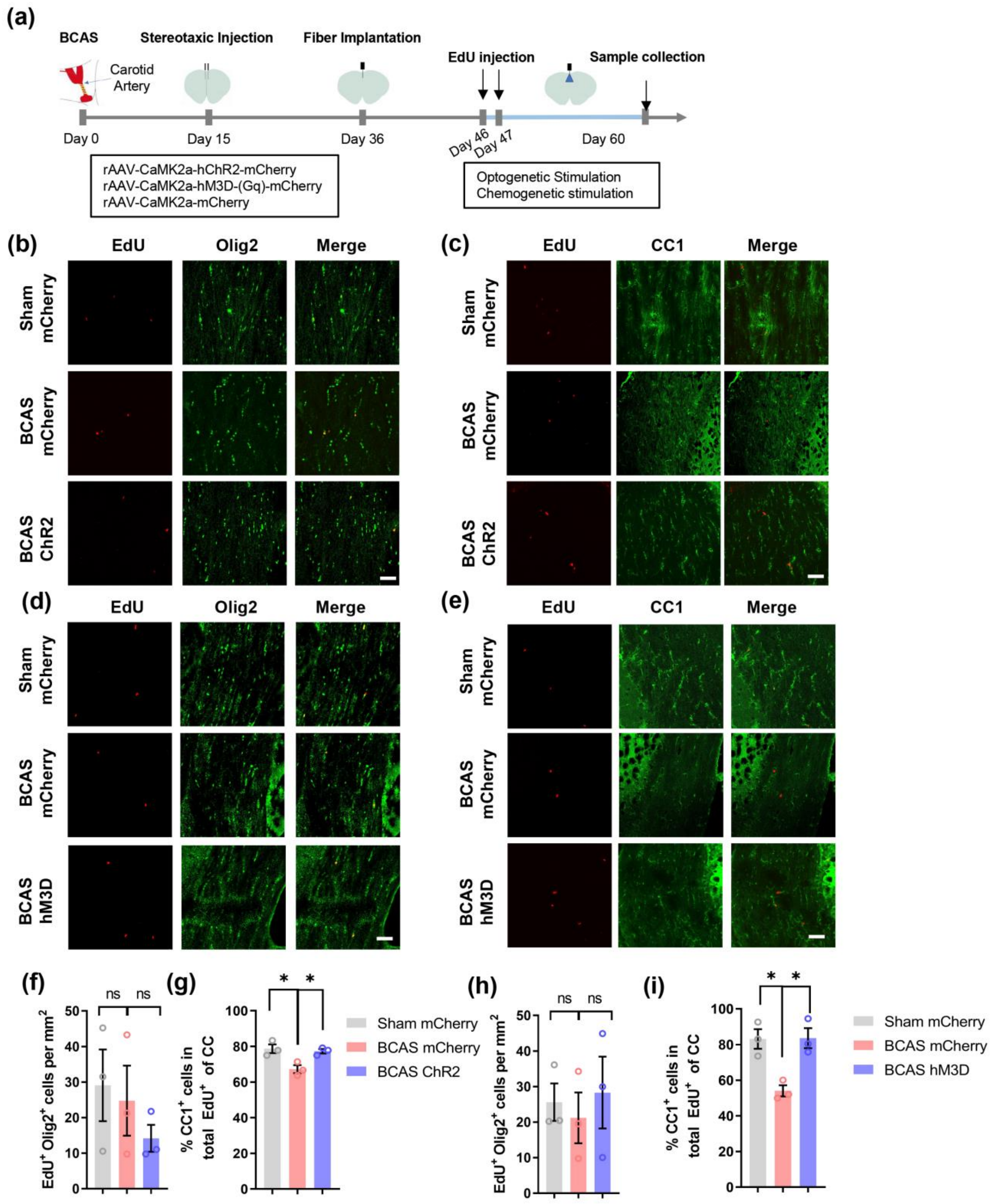

Figure S3. Effects of mPFC activation on oligodendrocyte lineage cells. a) Schematic diagram of 2-day EdU injection into BCAS model mice followed by long-term optogenetic or chemogenetic stimulation. b) Representative EdU staining and immunostaining of Olig2 in the corpus callosum in the sham mCherry, BCAS mCherry, and BCAS ChR2 groups at 2 months after surgery. Scale bar: 50 μm. c) Representative EdU staining and immunostaining

of CC1 in the corpus callosum in the sham mCherry, BCAS mCherry, and BCAS ChR2 groups at 2 months after surgery. Scale bar: 50 µm. d) Representative EdU staining and immunostaining of Olig2 in the corpus callosum in the sham mCherry, BCAS mCherry, and BCAS hM3D groups at 2 months after surgery. Scale bar: 50 µm. e) Representative EdU staining and immunostaining of CC1 in the corpus callosum in the sham mCherry, BCAS mCherry, and hM3D groups at 2 months after surgery. Scale bar: 50 µm. f) Density of EdU^+ $Olig2^+$ cells in the corpus callosum in the sham mCherry, BCAS mCherry, and BCAS ChR2 groups at 2 months after surgery. n=3 per group. g) Proportion of $CC1^+$ cells in total EdU^+ cells of corpus callosum in the sham mCherry, BCAS mCherry, and BCAS ChR2 groups at 2 months after surgery. n=3 per group. h) Density of EdU^+ $Olig2^+$ cells in the corpus callosum in the sham mCherry, BCAS mCherry, and BCAS hM3D groups at 2 months after surgery. n=3 per group. i) Proportion of $CC1^+$ cells in total EdU^+ cells of corpus callosum in the sham mCherry, BCAS mCherry, and BCAS hM3D groups at 2 months after surgery. n=3 per group. The data are presented as the mean ± SEM. P values were determined by the Kruskal–Wallis test with Dunn's post-hoc analysis in (h); by 1-way ANOVA with Tukey's post-hoc analysis in (f), (g), and (i). ns, $P > 0.05$, $*P < 0.05$.

Figure. S4

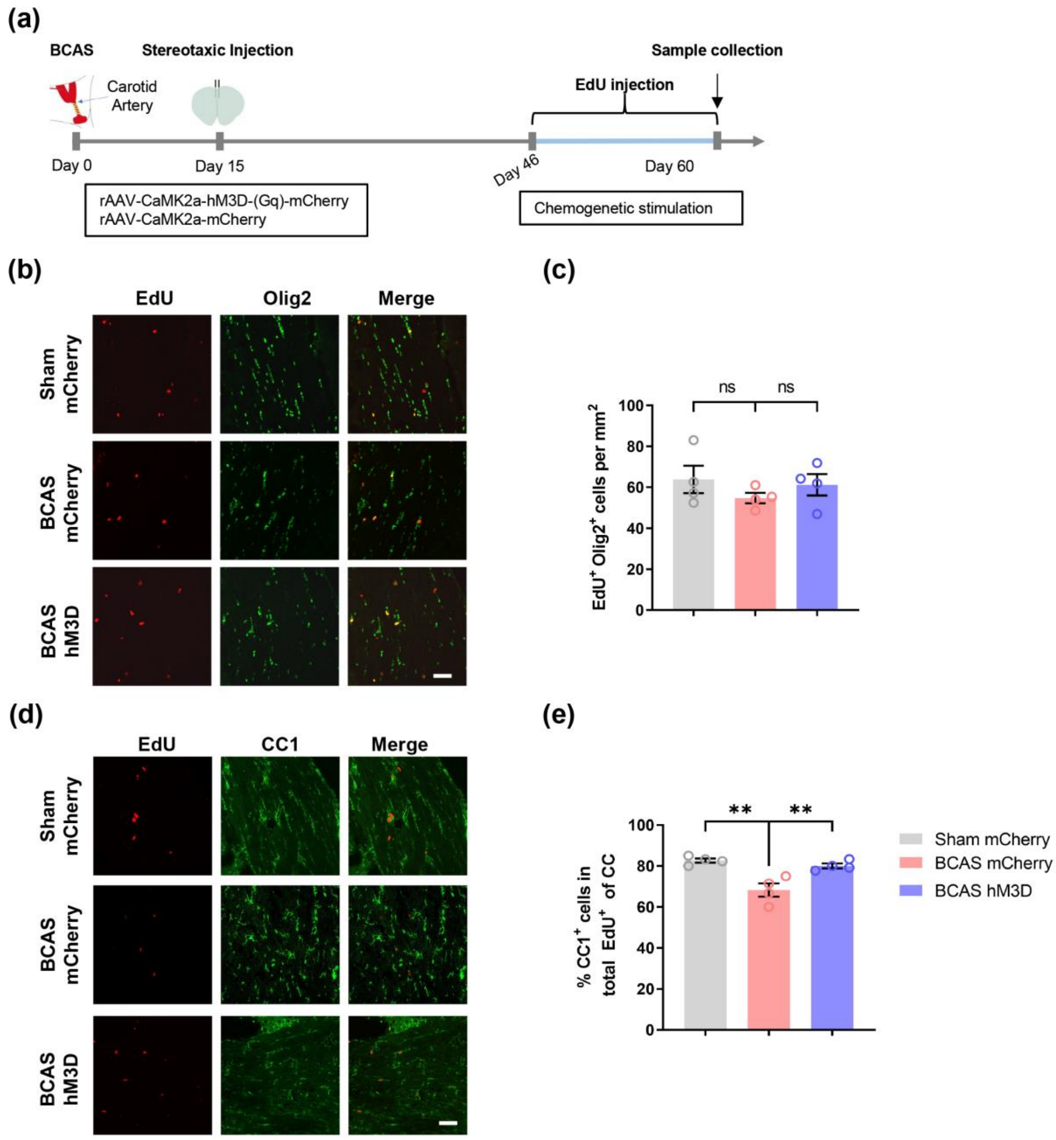

Figure S4. Chemogenetic stimulation of mPFC promotes oligodendrocyte differentiation. a) Schematic diagram of 14-day EdU injection into BCAS model mice followed by long-term chemogenetic stimulation. b) Representative EdU staining and immunostaining of Olig2 in the corpus callosum in the sham mCherry, BCAS mCherry, and BCAS hM3D groups at 2 months after surgery. Scale bar: 50 µm. c) Density of EdU^+ $Olig2^+$ cells in the corpus callosum in the sham mCherry, BCAS mCherry, and BCAS hM3D groups at 2 months after surgery. *n*=4 per group. d) Representative EdU staining and immunostaining of CC1 in the corpus callosum in the sham mCherry, BCAS mCherry, and BCAS ChR2 groups at 2 months

after surgery. Scale bar: 50 μm. e) Proportion of CC1^{+} cells in total EdU^{+} cells of corpus callosum in the sham mCherry, BCAS mCherry, and BCAS hM3D groups at 2 months after surgery. *n*=4 per group. The data are presented as the mean ± SEM. *P* values were determined by 1-way ANOVA with Tukey's post-hoc analysis in (c) and (e). ns, $P > 0.05$, $^{**}P < 0.01$.

Figure. S5

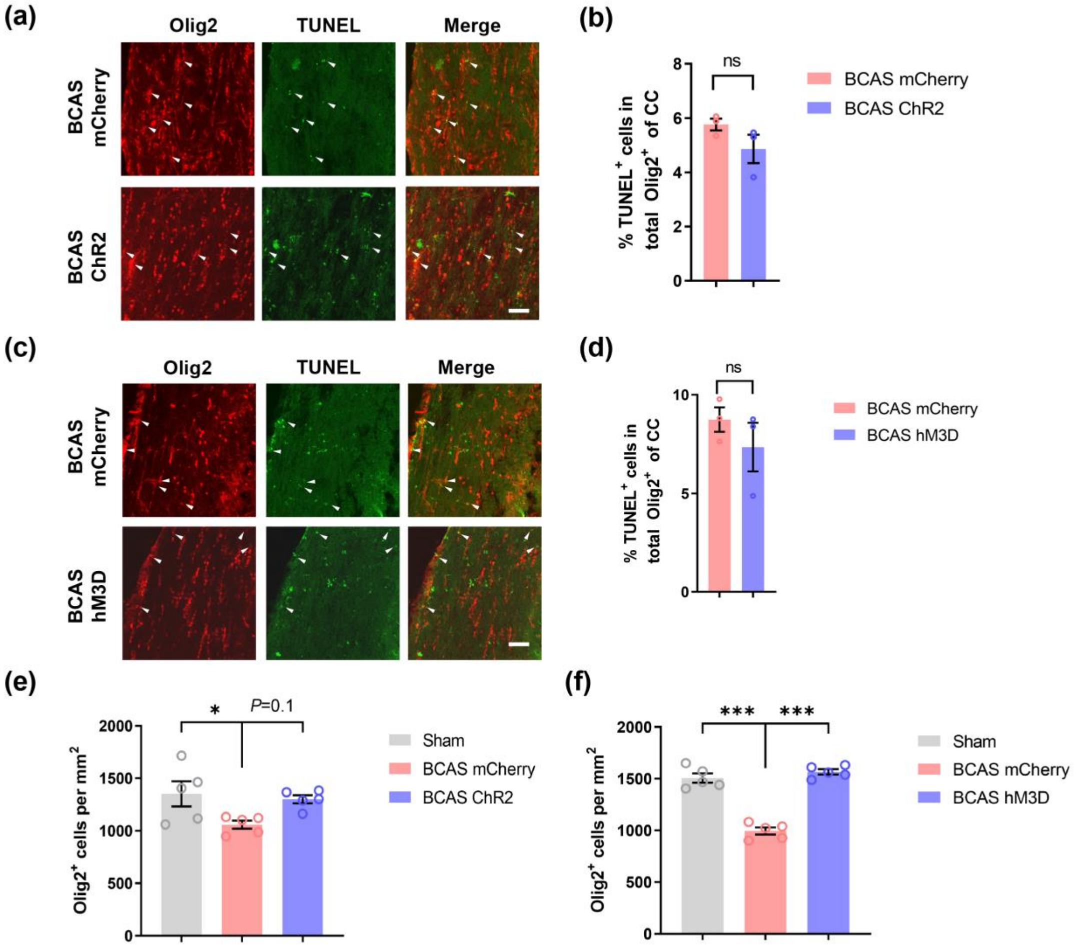

Figure S5. Neuromodulation of mPFC does not affect the survival of oligodendrocyte lineage cells. a) Representative TUNEL staining and immunostaining of Olig2 in the corpus callosum in the BCAS mCherry and BCAS ChR2 groups at 2 months after surgery. Scale bar: 50 μm. b) Proportion of TUNEL^{+}/Olig2^{+} cells in the corpus callosum in the BCAS mCherry and BCAS ChR2 groups at 2 months after surgery. *n*=3 per group. c) Representative TUNEL staining and immunostaining of Olig2 in the corpus callosum in the BCAS mCherry and BCAS hM3D

groups at 2 months after surgery. Scale bar: 50 μm. d) Proportion of TUNEL$^+$/Olig2$^+$ cells in the corpus callosum in the BCAS mCherry and BCAS hM3D groups at 2 months after surgery. *n*=3 per group. e) Density of Olig2$^+$ cells in the corpus callosum in the sham mCherry, BCAS mCherry, and BCAS ChR2 groups at 2 months after surgery. *n*=5 per group. f) Density of Olig2$^+$ cells in the corpus callosum in the sham mCherry, BCAS mCherry, and BCAS hM3D groups at 2 months after surgery. *n*=5 per group. The data are presented as the mean ± SEM. *P* values were determined by Student's t test in (b) and (d); and by 1-way ANOVA with Tukey's post-hoc analysis in (e) and (f). ns, $P > 0.05$, *$P < 0.05$, ***$P < 0.001$.

Figure. S6

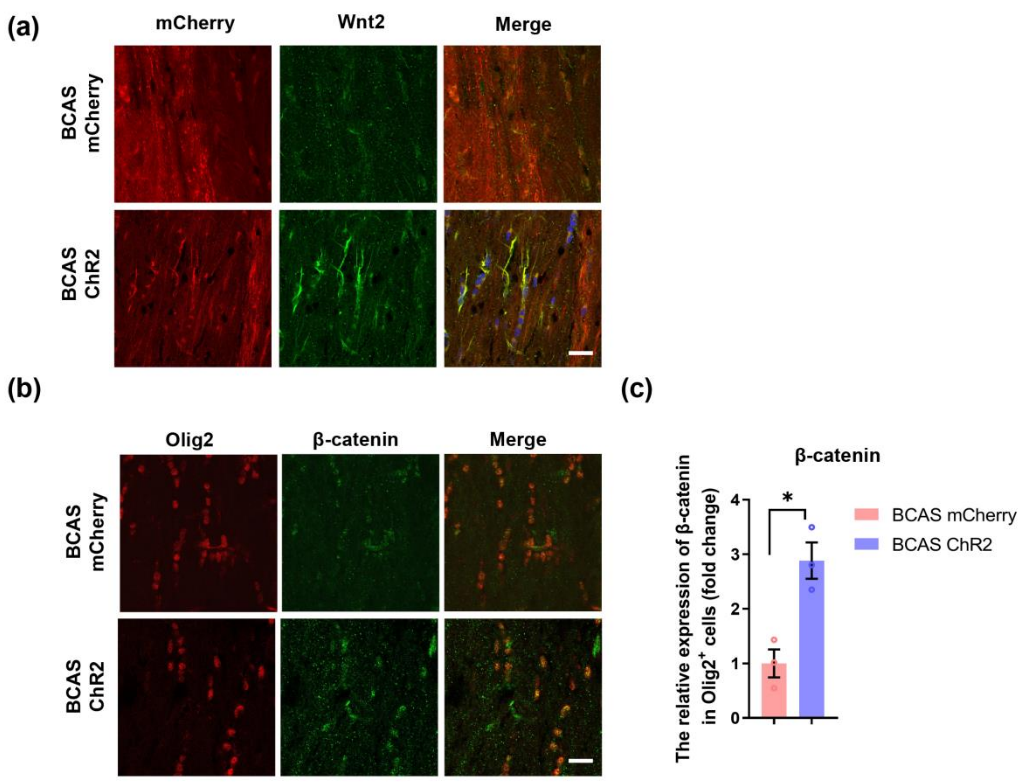

Figure S6. Activation of glutamatergic neurons in the mPFC upregulates the expression of Wnt2 and β-catenin in the corpus callosum. a) Representative immunostaining of Wnt2 and rAAV-associated mCherry expression (mCherry$^+$ axon) in the mPFC in the BCAS mCherry and BCAS ChR2 groups at 2 months after surgery. Scale bar: 35 μm. b) Representative

immunostaining of Olig2 and β-catenin in the corpus callosum in the BCAS mCherry and BCAS ChR2 groups at 2 months after surgery. Scale bar: 35 μm. c) Quantification of β-catenin immunostaining in the corpus callosum in the BCAS mCherry and BCAS ChR2 groups at 2 months after surgery. The fluorescence intensity in each image was normalized to that of Olig2. The measured values were normalized to the mean value of the BCAS group. *n*=3 per group. The data are presented as the mean ± SEM. *P* values were determined by Student's t test in (c). **P* < 0.05.

Figure. S7

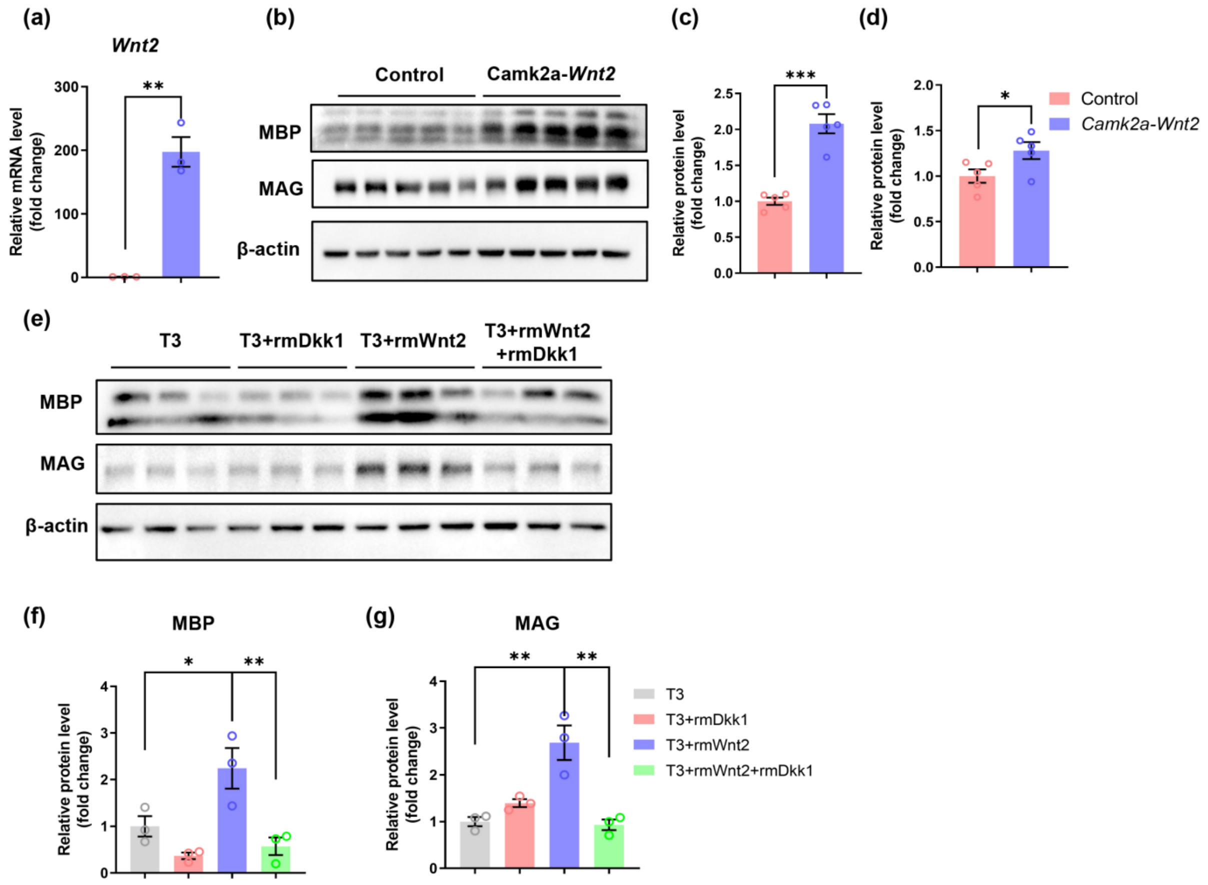

Figure S7. Wnt2 promotes oligodendrocyte differentiation. a) Real-time PCR analysis of *Wnt2* expression in the mPFC in the control and *Camk2a-Wnt2* groups at 2 months after

surgery. *n*=3 per group. b-d) Immunoblot bands (b) and quantification of MBP (c) and MAG (d) expressionin the control and *Camk2a-Wnt2* groups at 2 months after surgery. The intensity of each immunoblot band was normalized to that of the β-actin band. The measured values were normalized to the mean value of the sham group. *n*=5 per group. e-g) Immunoblot bands (e) and quantification of MBP (f) and MAG (g) expression in OPCs in the T3, T3+rmDkk1, T3+ rmWnt2, T3+rmDkk1+rmWnt2 groups. The intensity of each immunoblot band was normalized to that of the β-actin band. The measured values were normalized to the mean value of the T3 group. *n*=3 per group. The data are presented as the mean ± SEM. *P* values were determined by Student's t test in (a), (c), and (d), and by 1-way ANOVA with Tukey's post-hoc analysis in (f) and (g). *$P < 0.05$, **$P < 0.01$, ***$P < 0.001$.
